# Supplementary material for: Public acceptability of the UK Soft Drinks Industry Levy: repeat cross-sectional analysis of the International Food Policy Study (2017–2019)
Source: BMJ Open. 2021 Sep 24;11(9):e051677. doi: 10.1136/bmjopen-2021-051677 (PMC8475144; doi:10.1136/bmjopen-2021-051677)
Supplement: Author's manuscript [file bmjopen-2021-051677.draft_revisions.pdf]

BMJ Open is committed to open peer review. As part of this commitment we make the peer review history of every article we publish publicly available.

When an article is published we post the peer reviewers' comments and the authors' responses online. We also post the versions of the paper that were used during peer review. These are the versions that the peer review comments apply to.

The versions of the paper that follow are the versions that were submitted during the peer review process. They are not the versions of record or the final published versions. They should not be cited or distributed as the published version of this manuscript.

BMJ Open is an open access journal and the full, final, typeset and author-corrected version of record of the manuscript is available on our site with no access controls, subscription charges or pay-per-view fees (http://bmjopen.bmj.com).

If you have any questions on BMJ Open's open peer review process please email info.bmjopen@bmj.com

CHANGE IN PUBLIC ACCEPTABILITY OF THE UK SOFT DRINKS INDUSTRY LEVY IN UK ADULTS FROM BEFORE TO AFTER IMPLEMENTATION: REPEAT CROSS-SECTIONAL ANALYSIS OF THE INTERNATIONAL FOOD POLICY STUDY (2017-2019)

Journal:	*BMJ Open*
Manuscript ID	bmjopen-2021-051677
Article Type:	Original research
Date Submitted by the Author:	12-Apr-2021

Complete List of Authors:	Adams, J; University of Cambridge, MRC Epidemiology Unit<br>Pell, David; University of Cambridge, MRC Epidemiology Unit; NHS RightCare, Midlands Region<br>Penney, Tarra; York University Faculty of Health, School of Global Health<br>Hammond, D; University of Waterloo, School of Public Health<br>Vanderlee, Lana; Universite Laval, Ecole de Nutrition<br>White, Martin; University of Cambridge, MRC Epidemiology Unit

Keywords:	NUTRITION & DIETETICS, PUBLIC HEALTH, Health policy < HEALTH SERVICES ADMINISTRATION & MANAGEMENT

SCHOLARONE™ Manuscripts

I, the Submitting Author has the right to grant and does grant on behalf of all authors of the Work (as defined in the below author licence), an exclusive licence and/or a non-exclusive licence for contributions from authors who are: i) UK Crown employees; ii) where BMJ has agreed a CC-BY licence shall apply, and/or iii) in accordance with the terms applicable for US Federal Government officers or employees acting as part of their official duties; on a worldwide, perpetual, irrevocable, royalty-free basis to BMJ Publishing Group Ltd ("BMJ") its licensees and where the relevant Journal is co-owned by BMJ to the co-owners of the Journal, to publish the Work in this journal and any other BMJ products and to exploit all rights, as set out in our licence.

The Submitting Author accepts and understands that any supply made under these terms is made by BMJ to the Submitting Author unless you are acting as an employee on behalf of your employer or a postgraduate student of an affiliated institution which is paying any applicable article publishing charge ("APC") for Open Access articles. Where the Submitting Author wishes to make the Work available on an Open Access basis (and intends to pay the relevant APC), the terms of reuse of such Open Access shall be governed by a Creative Commons licence – details of these licences and which Creative Commons licence will apply to this Work are set out in our licence referred to above.

Other than as permitted in any relevant BMJ Author's Self Archiving Policies, I confirm this Work has not been accepted for publication elsewhere, is not being considered for publication elsewhere and does not duplicate material already published. I confirm all authors consent to publication of this Work and authorise the granting of this licence.

For peer review only

CHANGE IN PUBLIC ACCEPTABILITY OF THE UK SOFT DRINKS INDUSTRY LEVY IN UK ADULTS FROM BEFORE TO AFTER IMPLEMENTATION: REPEAT CROSS-SECTIONAL ANALYSIS OF THE INTERNATIONAL FOOD POLICY STUDY (2017-2019)

Jean Adams,[1] David Pell,[1,2†] Tarra Penney,[3] David Hammond,[4] Lana Vanderlee,[5] Martin White[1]*

[1]Centre for Diet & Activity Research, MRC Epidemiology Unit, University of Cambridge, Cambridge, UK

[2]RightCare, Midlands Region, NHS England, Newcastle upon Tyne, UK

[3]School of Global Health, York University, Toronto, Ontario, Canada

[4]School of Public Health, University of Waterloo, Waterloo, Ontario, Canada

[5]Centre NUTRISS (Nutrition, Santé et Société), École de Nutrition, Université Laval, Quebec, Quebec, Canada

[†]DP was affiliation 1 when this work was originally conceived, but is now affiliation 2

***Corresponding author:** Jean Adams, Centre for Diet & Activity Research, MRC Epidemiology Unit, University of Cambridge School of Clinical Medicine, Box 285 Institute of Metabolic Science, Cambridge Biomedical Campus, Cambridge, UK, CB2 0QQ. Email: jma79@medschl.cam.ac.uk Tel: +44 (0) 1223 769 142

Main text word count: 3493

For peer review only

ABSTRACT

Objectives To determine whether public acceptability, in terms of both support for and perceived effectiveness of, the UK Soft Drinks Industry Levy (SDIL) changed between four months prior to, and 8 and 20 months after, implementation.

Design Repeat cross-sectional on-line survey.

Setting UK

Participants UK respondents to the International Food Policy Study aged 18-64 years who provided information on all variables of interest in November-December 2017 (4 months prior to SDIL implementation), 2018 (8 months after) or 2019 (20 months after; n=10,284).

Outcome measures Self-reported support for, and perceived effectiveness of, the SDIL.

Results The adjusted logistic regression model predicted that 70% (95% CI: 68 to 72) of participants supported the SDIL in 2017, 68% (95% CI: 67 to 70) in 2018, and 68% (95% CI: 66 to 70) in 2019. There was no evidence of a difference in support in 2018 vs 2017 (odds ratio (OR): 0.93; 95% confidence intervals (CI): 0.81 to 1.05); or in 2019 vs 2017 (OR: 0.90; 95% CI (0.78 to 1.03)). The adjusted logistic regression model predicted that 72% (95% CI: 70 to 74) of participants perceived the SDIL to be effective in 2017, 67% (95% CI: 65 to 69) in 2018, and 67% (95% CI: 64 to 69) in 2019. There was evidence that perceived effectiveness decreased a small amount between 2018 vs 2017 (OR: 0.78; 95% CI: 0.69 to 0.88). The difference in 2019 vs 2017 was similar.

Conclusions There was high support for the SDIL amongst UK adults and this did not change between 4 months before implementation and 8 or 20 months after. Whilst perceived effectiveness remained high, there was evidence that this decreased slightly after implementation in 2018, but no further in 2019. Greater understanding of influences on public acceptability of effective structural public health interventions is required.

Keywords: taxation, soda tax, public health, attitudes, nutrition & dietetics

For peer review only - http://bmjopen.bmj.com/site/about/guidelines.xhtml

ARTICLE SUMMARY

Strengths and limitations of the study

- We used three annual waves of a large, population-based survey (n=10,284).
- We were careful to present the Soft Drinks Industry Levy as an intervention targeted at manufacturers rather than consumers, with revenues ear-marked for health-promotion activities.
- This is a repeat cross-sectional design with measures pre- and post-implementation
- Whilst all measures have strong face validity, we have not explored other aspects of validity or reliability of any of the measures used; in many cases it would be hard to know what the 'gold standard' measure should be.

For peer review only

For peer review only - http://bmjopen.bmj.com/site/about/guidelines.xhtml

INTRODUCTION

Taxes on sugar sweetened beverages (SSBs) are recommended by the World Health Organisation to reduce sugar consumption and prevent non-communicable diseases.[1] Systematic review evidence suggests that SSB taxes lead to reductions in SSB purchasing and consumption, but there is substantial heterogeneity in effect sizes[2] and tax design.[3] Better understanding of the contextual factors that influence the effectiveness of SSB taxes may enable taxes to be better tailored to context.[4] Such contextual factors may include public acceptability of SSB taxes.

By their nature, regulatory policies such as SSB taxes require political support for implementation. Political support is, in turn, likely to be influenced by public acceptability. As well as influencing implementation, public acceptability may also influence the effectiveness and longevity of SSB taxes.[5] For example, if price increases following an SSB tax are not acceptable to the public, then they may travel to buy SSBs in un-taxed areas (so called cross-border shopping).[3] Further, a number of food taxes have been repealed after implementation, in part due to public backlash.[6-8] This makes it important to understand how public acceptability of SSB taxes changes after implementation.

Public acceptability of a policy reflects both public support for that policy and perceptions of how effective the policy may be.[9] Public support for hypothetical SSB taxes ranges from around 35-60%.[10-27] A recent systematic review reported a pooled figure for support of 42% (95% confidence interval (CI): 38 to 47).[9] Associations between support and variables such as age, SSB consumption and socio-economic position are inconsistent.[10 12 15 17 23-25 27-29] However, support is consistently higher when it is clear that revenue raised will be used for health promotion activities, such as subsidies on healthy food.[9 18 19 26 29 30]

Perceived effectiveness of SSB taxes has been less studied than public support.[9 17 29] However, in a systematic review, pooled estimates were that 39% (95% CI 26 to 54) of the public believe SSB taxes reduce purchases and consumption, and 40% (95% CI 29 to 54) believe that they impact on health-related outcomes.[9] A perception that SSB taxes are unlikely to be effective is a common explanation for low public support in qualitative studies.[9 16 18]

Most previous work on public acceptability of SSB taxes has focused on hypothetical taxes. A systematic review of support for government interventions to change health-related behaviours (that did not include any studies on SSB taxes) found that support tends to be higher for implemented, rather than

For peer review only - http://bmjopen.bmj.com/site/about/guidelines.xhtml

hypothetical, policies.[31] Similarly, perceived effectiveness may be influenced by whether respondents are reporting on a hypothetical or implemented tax.

We are aware of four studies on public acceptability of implemented SSB taxes. One study of the French excise tax on sweetened beverages found 49% of the public supported the tax and 58% believed it would improve health.[29] An international study exploring public support for a range of dietary public health policies in 2017 included data from Mexico, where an SSB tax was implemented in 2014. Whilst support for an SSB tax was higher in Mexico than other countries (54% vs 30-49%), the same was true for many other policies studied.[27] Two studies have focused on the UK Soft Drinks Industry Levy (SDIL; described in Box 1).[32 33] Our previous population-based survey conducted before implementation (i.e. the 2017 data presented in the current work), found that 70% of UK adults supported the SDIL and 71% thought it would be effective.[32] Finally, a survey of parents of children aged 5-11 years conducted soon after implementation found that 57% supported the aims of the SDIL.[33] We are not aware of any study exploring change in public acceptability of SSB taxes from before to after implementation.

Our aim was to determine whether public acceptability, in terms of both support for, and perceived effectiveness of, the SDIL changed between four months prior to and 20 months after implementation.

Box 1 – Key characteristics of the UK Soft Drinks Industry Levy[34-37]

- Levied on companies importing or manufacturing SSBs, not consumers
- Intentional two-year delay between announcement (March 2016) and implementation (April 2018) to give manufacturers time to adapt by developing lower sugar products
- Tiered with eligible drinks containing ≥8g of sugar per 100ml charged £0.24 [€0.27, US$0.33]/litre, those containing ≥5g but <8g charged £0.18 [€0.20, US$0.24]/litre, and those containing <5g not charged
- Exemptions for pure fruit juices, milk-based drinks and a number of other smaller categories
- Announcement included a statement that revenue raised would be spent on school sport and school breakfast clubs
- Associated with substantial reformulation of the UK soft drinks market to reduce sugar content
- Associated with complex changes in SSB prices with some categories increasing in price and others decreasing
- Associated with no change in volume of all drinks purchased, but a reduction in sugar purchased from drinks

For peer review only - http://bmjopen.bmj.com/site/about/guidelines.xhtml

METHODS

We used repeat cross-sectional survey data from the International Food Policy Study (IFPS). We conducted both unadjusted analyses and analyses adjusted for a number of socio-demographic and psychological variables that are potential, or previously reported, correlates of support for, and perceived effectiveness of, the SDIL.

Sampling, recruitment and data collection

Data were from UK participants in the 2017, 2018 and 2019 waves of IFPS. This is an annual repeat cross-sectional survey conducted in Australia, Canada, Mexico, the UK and the USA with an annual, pragmatic, recruitment target of 4000 adults per country per wave. Data were collected via self-completed web surveys in November-December each year, representing 4-5 months before implementation, but 19-20 months after announcement (2017); 7-8 months after implementation (2018) and 19-20 months after implementation (2019). Respondents were recruited through Nielsen Consumer Insights Global Panel and their partners' panels. Email invitations with unique survey access links were sent to a random sample of panellists within each country after targeting for demographics; panellists known to be ineligible were not invited. Potential respondents were screened for eligibility and quota requirements based on age and sex.

Respondents provided informed consent prior to completing the survey and received remuneration in accordance with their panel's usual incentive structure (e.g. points-based or monetary rewards, or chances to win prizes). A full description of the study methods can be found at www.foodpolicystudy.com/methods.

Inclusion criteria

We included in the analysis UK resident participants in the 2017, 2018 and 2019 IFPS waves who met the following criteria: were aged 18-64 years, provided information on sex at birth and age, and passed data quality checks (provided a valid response to a data quality question; took at least 15 minutes to complete the survey; and provided a valid response to at least three of 20 open-ended measures).

Variables used in the analysis

The variables used in the analysis, the survey items from which they were derived, response options and how response options were collapsed for analysis are described in Table 1.

For peer review only - http://bmjopen.bmj.com/site/about/guidelines.xhtml

Table 1. Description of items and response options used in the analysis

Concept	Item wording (where applicable)	Response options: All	Response options: Used in analysis
Age	How old are you?	In years	In years
Sex	What sex were you assigned at birth, meaning on your original birth certificate?	Female	Female
		Male	Male
Education	What is the highest level of education you have completed?	Qualifications not listed below, free-text equivalents, Don't Know, Refuse to answer	School level
		NVQ Level 4-5, HNC, HND, RSA Higher Diploma, BTEC Higher Level, Degree, Higher Degree, free-text equivalents	Post school level
Income sufficiency	How easy is it to make ends meet?	Neither easy nor difficult, Difficult, Very difficult, Don't know, Refuse to answer	Not easy
		Very easy, Easy	Easy
Children	Do you have any children (including step-children or adopted children) under the age of 18?	No, Don't know, Refuse to answer	No
		Yes	Yes
SSB consumption	[Calculated from Beverage Frequency Questionnaire: reported consumption over last 7 days]	Any consumption of non-diet Fizzy drinks, Sweetened fruit juice drinks, Regular sports drinks, Regular energy drinks, or Spirits with mixers that have calories	Consumers
		No consumption of above	Non-consumers
Social norms	People important to me try not to drink sugary drinks	Neither agree nor disagree, Disagree, Strongly disagree, Don't know, Refuse to answer	Not agree
		Strongly agree, Agree	Agree
Attitudes	Sugary drinks taste good	Strongly agree, Agree	Agree
		Neither agree nor disagree, Disagree, Strongly disagree, Don't know, Refuse to answer	Not agree
Knowledge	Frequently drinking sugary drinks increases the risk of obesity	False, Don't know, Refuse to answer	Not true
		True	True
Expert trust	I trust messages from health experts on sugary drinks	Neither agree nor disagree, Disagree, Strongly disagree, Don't know, Refuse to answer	Not agree
		Strongly agree, Agree	Agree
Industry trust	I trust messages from the food and beverage industry on sugary drinks	Neither agree nor disagree, Disagree, Strongly disagree, Don't know, Refuse to answer	Not agree
		Strongly agree, Agree	Agree
Support	In 2018 a new sugary drink tax will be/was introduced in the UK. This aims to encourage manufacturers to reduce the sugar in drinks. The money will be spent on breakfast clubs, and sports in primary schools. Do you support or oppose this policy?	Strongly support, Support	Support
		Oppose, Strongly oppose, Don't know, Refuse to answer	Oppose
Effectiveness	Preamble as above. How effective do you think these kinds of policies would be/are?	Somewhat effective, Mostly effective, Very effective	Effective
		Not at all effective, Don't know, Refuse to answer	Not effective

Note. SSB = sugar sweetened beverage; NVQ = National Vocational Qualification; HNC = Higher National Certificate; HND = Higher National Diploma, RSA = Royal Society of Arts; BTEC = Business and Technology Education Council

For peer review only - http://bmjopen.bmj.com/site/about/guidelines.xhtml
For peer review only

Outcome variables

The outcome variables of interest were single-item measures of support for, and perceived effectiveness of, the SDIL as described in Table 1.

Exposure variable

The exposure variable of interest was year – either 2017, 2018 or 2019.

Potential confounding variables

We adjusted for a number of individual-level socio-demographic and psychological variables in the analysis (see Table 1 for details). With the exception of age, education and income sufficiency, these all showed associations with one or both outcome variables in our previous analysis of 2017 data.[32] Given that age and markers of socio-economic position, such as education and income sufficiency, have been previously, if inconsistently, associated with public support for SSB taxes,[10 12 15 17 23-25 28 29] we included them here, despite no relationship with the outcomes in our previous analysis.

We included single-item measures of attitudes, knowledge, and social norms related to sugary drinks; and trust in advice on sugary drinks from health experts and the food and beverage industry. As previous research has indicated that the acceptability of food taxes varies with the stated intentions of these,[9 18 19 26 29 30] we included a preamble to the questions about support for, and perceived effectiveness of, the SDIL outlining the intention of the levy and the stated use of revenue generated.

Socio-demographic variables considered were age in years, sex at birth, whether or not participants had dependent children, and socio-economic position. Parental status was included as the SDIL has been particularly framed in terms of potential benefits for children.[35 38] Socio-economic position was measured using participants' highest educational qualification and perceived income sufficiency.

Current behaviour has previously been associated with perceived acceptability of public health interventions,[31] and we found that SSB consumers were less likely to support the SDIL in 2017. As such, we adjusted for SSB consumption using the Beverage Frequency Questionnaire. This is a 7-day food record that assesses consumption of 17 beverage categories, including caloric and non-caloric beverages.[39] For each beverage category, respondents report the number of drinks and the usual portion size using category-specific images of beverage containers adapted from the Automated Self-Administered 24 hour (ASA24) dietary assessment tool. Participants who reported any consumption of regular fizzy drinks, alcoholic drinks with regular mixers or cocktails that have calories, sweetened fruit drinks, sports drinks, or energy drinks over the previous seven days were considered SSB consumers.

For peer review only - http://bmjopen.bmj.com/site/about/guidelines.xhtml

Analysis

Data were weighted with post-stratification sample weights constructed using population estimates from the UK census based on age group, sex and region. These were used throughout the analysis to reduce the effects of non-response and selection bias.

Descriptive statistics were used to quantify all variables of interest. Logistic regression models were fitted to explore associations between study wave and support for, and perceived effectiveness of, the SDIL before and after adjustment for other variables. We used separate models to explore support for the SDIL and perceived effectiveness of the SDIL. In these models support or perceived effectiveness were the outcome variables, study wave was the exposure variable, and all other variables were covariates. We used the fully adjusted models, and mean values of covariates, to predict the proportion of the population likely to be supportive of the SDIL, and think it would be effective, at each time point.

Data were analysed using Stata version 15.

Ethics

The study received ethical clearance from a University of Waterloo Research Ethics Committee (ORE# 21460 and ORE# 30829). All participants provided informed consent to take part.

Patient and public involvement

Patients and the public were not involved in the design, conduct, analysis or interpretation of the study.

RESULTS

A total of 25,692 adults took part in IFPS across all included countries in 2017, 28,684 in 2018 and 29,290 in 2019. After removing respondents with missing data on sex at birth and age, and those who did not meet data quality checks, 18,878 (73.5%) respondents remained in 2017, 22,824 (79.6%) in 2017 and 20,968 (71.6%) in 2019. Of these, 4047 were from the UK in 2017, 5549 in 2018, and 4139 in 2019. Amongst these UK participants, 3104 (76.7%) met the additional inclusion criteria for the current work in 2017, 4118 (74.2%) in 2018, and 3062 (74.0%) in 2019. Characteristics of the analytical sample (after applying survey weights) are described in Table 2. Mean age (standard deviation) was 38 (13) years in 2107 participants, 41 (13) in 2018, and 42 (13) in 2019.

For peer review only - http://bmjopen.bmj.com/site/about/guidelines.xhtml

Table 2. Weighted unadjusted characteristics of UK participants; International Food Policy Study, 2017-19

Concept	Question wording	Response category	Weighted percentage (95% confidence intervals)			
			2017, n=3104	2018, n=4118	2019, n=3062	Total, n=10,284
Sex	What sex were you assigned at birth, meaning on your original birth certificate?	Female	48 (46 to 50)	50 (48 to 52)	50 (48 to 52)	49 (48 to 51)
Education	What is the highest level of education you have completed?	School level	61 (59 to 63)	74 (72 to 75)	74 (73 to 76)	70 (69 to 71)
Income sufficiency	How easy is it to make ends meet?	Not easy	61 (59 to 63)	66 (65 to 68)	66 (64 to 68)	65 (64 to 66)
Children	Do you have any children (including step-children or adopted) under 18?	No	63 (61 to 65)	68 (66 to 70)	65 (62 to 67)	66 (64 to 67)
SSB consumption	Consumed regular fizzy drinks, sweetened fruit drinks, sports drinks, energy drinks in last week	Consumers	53 (50 to 55)	44 (42 to 45)	44 (42 to 47)	46 (45 to 48)
Social norms	People important to me try not to drink sugary drinks	Not agree	46 (44 to 48)	52 (50 to 54)	51 (48 to 53)	50 (48 to 51)
Attitudes	Sugary drinks taste good	Agree	62 (60 to 64)	64 (63 to 66)	59 (57 to 61)	62 (61 to 63)
Knowledge	Frequently drinking sugary drinks increases the risk of obesity	Not true	10 (9 to 12)	14 (13 to 16)	12 (10 to 13)	12 (12 to 13)
Expert trust	I trust messages from health experts on sugary drinks	Not agree	39 (37 to 41)	40 (38 to 42)	41 (39 to 43)	40 (39 to 41)
Industry trust	I trust messages from the food and beverage industry on sugary drinks	Not agree	73 (71 to 75)	69 (67 to 70)	68 (66 to 70)	70 (69 to 71)
Support	In 2018 a new sugary drink tax will be/was introduced in the UK. This aims to encourage manufacturers to reduce the sugar in drinks. The money will be spent on breakfast clubs, and sports in primary schools. Do you support or oppose this policy?	Support	70 (68 to 72)	66 (64 to 68)	66 (64 to 68)	67 (66 to 68)
Effectiveness	Preamble as above. How effective do you think these kinds of policies are?	Effective	71 (69 to 73)	66 (64 to 67)	65 (63 to 67)	67 (66 to 68)

Note. SSB = sugar sweetened beverage

For peer review only

For peer review only - http://bmjopen.bmj.com/site/about/guidelines.xhtml

Table 3 shows the results of logistic regression analyses of associations between survey wave and support for, and perceived effectiveness of, the SDIL, before and after adjusting for the socio-demographic and psychological concepts listed. In unadjusted analyses, both support for, and perceived effectiveness of, the SDIL dropped between 2017 and 2018, but there was little difference in effect estimates in 2018 vs 2017 and in 2019 vs 2017.

In adjusted analyses there was no evidence that the proportion of participants supporting the SDIL changed between 2017 and either 2018 or 2019. In contrast, the proportion who perceived the SDIL to be effective in 2018 and 2019 was lower than that in 2017. However, the difference in the proportion who perceived the SDIL to be effective was very similar in 2018 vs 2017 and 2019 vs 2017 indicating that the decreased in perceived effectiveness occurred between 2017 and 2018.

Holding all other variables at their mean levels, the adjusted logistic regression model predicted that 70% (95% CI: 68 to 72) of participants supported the SDIL in 2017, 68% (95% CI: 67 to 70%) in 2018, and 68% (95% CI: 66 to 70) in 2019 (Figure 1). Comparable figures for perceived effectiveness were 72% (95% CI: 70 to 74) in 2017, 67% (95% CI: 65 to 69) in 2018, and 67% (95% CI: 64 to 69) in 2019 (Figure 1).

Other variables in the adjusted models were also associated with support for, and perceived effectiveness of, the SDIL (Table 3). Greater support for the SDIL was associated with: older age, having a higher level of education, not having children at home, being a non-consumer of SSBs, having social norms to avoid sugary drinks, disliking the taste of sugary drinks, recognising an association between sugary drinks and obesity, trusting health expert messages on sugary drinks and not trusting industry messages on sugary drinks. Greater perceived effectiveness of the SDIL was associated with: younger age, having social norms to avoid sugary drinks, disliking the taste of sugary drinks, trusting health expert messages on sugary drinks and trusting industry messages on sugary drinks.

For peer review only

For peer review only - http://bmjopen.bmj.com/site/about/guidelines.xhtml

Table 3. Adjusted odds ratios (95% confidence intervals) of the association between year and support for, and perceived effectiveness of, the Soft Drinks Industry Levy; International Food Policy Study, 2017-19

			Support for the SDIL		Perceived effectiveness of the SDIL	
**Concept**	**Question wording (where applicable)**	**Response category**	**Unadjusted**	**Adjusted**	**Unadjusted**	**Adjusted**
Survey wave	Not applicable	2017	Reference	Reference	Reference	Reference
		2018	**0.84 (0.75 to 0.95)**	0.93 (0.81 to 1.05)	**0.77 (0.68 to 0.87)**	**0.78 (0.69 to 0.88)**
		2019	**0.84 (0.74 to 0.96)**	0.90 (0.78 to 1.03)	**0.76 (0.67 to 0.87)**	**0.76 (0.66 to 0.86)**
Age	How old are you?	Years		**1.02 (1.01 to 1.02)**		**0.98 (0.98 to 0.98)**
Sex	What sex were you assigned at birth, meaning on your original birth certificate?	Female		Reference		Reference
		Male		1.02 (0.92 to 1.13)		1.04 (0.94 to 1.15)
Education	What is the highest level of education you have completed?	School level		Reference		Reference
		Post school level		**1.19 (1.07 to 1.32)**		1.00 (0.90 to 1.10)
Income sufficiency	How easy is it to make ends meet?	Not easy		Reference		Reference
		Easy		1.07 (0.95 to 1.19)		1.04 (0.93 to 1.16)
Dependent children	Do you have any children (including step-children or adopted children) under 18?	No		Reference		Reference
		Yes		**0.81 (0.73 to 0.91)**		1.11 (0.99 to 1.24)
SSB consumption	Consumed regular fizzy drinks, sweetened fruit drinks, sports drinks, energy drinks in last week	Consumers		Reference		Reference
		Non-consumers		**1.12 (1.01 to 1.25)**		1.01 (0.91 to 1.12)
Social norms	People important to me try not to drink sugary drinks	Not agree		Reference		Reference
		Agree		**1.35 (1.21 to 1.50)**		**1.35 (1.21 to 1.51)**
Attitudes	Sugary drinks taste good	Agree		Reference		Reference
		Not agree		**1.32 (1.18 to 1.48)**		**1.34 (1.21 to 1.50)**
Knowledge	Frequently drinking sugary drinks increases the risk of obesity	Not true		Reference		Reference
		True		**2.76 (2.35 to 3.25)**		**1.26 (1.07 to 1.48)**
Expert trust	I trust messages from health experts on sugary drinks	Not agree		Reference		Reference
		Agree		**2.36 (2.09 to 2.66)**		**1.96 (1.76 to 2.20)**
Industry trust	I trust messages from the food and beverage industry on sugary drinks	Not agree		Reference		Reference
		Agree		**0.72 (0.64 to 0.82)**		**1.52 (1.34 to 1.73)**

Note. SDIL = Soft Drinks Industry Levy; **BOLD** indicates statistically significant at the p<0.05 level; adjusted for all concepts listed

For peer review only - http://bmjopen.bmj.com/site/about/guidelines.xhtml
For peer review only

DISCUSSION

Summary of findings

As far as we are aware, this is the first study to explore whether public acceptability of an SSB tax, operationalised in terms of support and perceived effectiveness, changed from before to after implementation. It also adds to the small existing literature on public acceptability of implemented (rather than hypothetical) SSB taxes. In this population-based, repeat cross-sectional survey, after adjustment for a range of socio-demographic and psychological covariates, we found that predicted support for the SDIL remained consistently high throughout (68-70%), with no evidence that support changed from four months before to 20 months after implementation. Whilst perceived effectiveness of the SDIL was also high throughout (67-72%), there was evidence that predicted perceived effectiveness of the SDIL decreased from 72% before implementation to 67% after implementation. This change was evident 7-8 months after implementation, with no further decrease 12 months later.

Strengths and weaknesses of methods

Key strengths of the analysis are the large (relatively to other work in the field)[9], population-based, sample; inclusion of a range of socio-demographic, consumption and psychological variables; the context of an implemented, rather than hypothetical, SSB tax in the latter two time points; and consistency of methods across all three time points. Given previous findings showing that support for SSB taxes is greater when revenues are used for health-promoting activities,[9 18 19 26 29 30] we were careful to present the SDIL with revenues ear-marked for health-promotion activities. We also clearly stated that it was an intervention designed to target manufacturers rather than consumers. Social desirability bias may also be less likely to occur in more anonymous settings such as on-line surveys.[40]

Participants were recruited using non-probability sampling. Despite the use of weights for age, sex and region, the findings do not necessarily provide nationally representative estimates; and are limited to ages 18-64 years. Whilst the pattern of results in terms of patterns of associations between variables are likely to be generalizable to the UK, the estimates of absolute frequency may not be. Given international differences in dietary public health policy,[41] the pattern of findings may not be generalizable beyond the UK. Although the IFPS takes place in a number of countries key questions used here were only asked of UK participants.

For peer review only - http://bmjopen.bmj.com/site/about/guidelines.xhtml

All variables were self-reported. Whilst all have strong face validity and the Beverage Frequency Questionnaire performs well compared to a seven day food record,[39] we have not explored validity or reliability of the other measures used. However, many were derived from existing instruments.

Comparison to previous results and interpretation of findings

Overall, there was high support for the SDIL throughout with no evidence of significant change across years. The 68-70% prevalence of support we found is noticeably higher than the previously reported ranges of 35-60%[10-26] and a pooled estimate of 42%.[9] We propose three potential reasons why support here may be higher than previously reported. Firstly, most previous data on support for SSB taxes has been collected in the context of hypothetical taxes. Previous systematic review evidence suggests that support for governmental public health interventions tends to increase after implementation.[31] Whilst 2017 data was collected four months prior to implementation of the SDIL, the policy intention was announced in March 2016, 20 months before 2017 data collection. As such, many participants may have either believed the SDIL had already been implemented, or at least accepted that it was going to be implemented, at the time of 2017 data collection. Comparable pre-announcement data is not available.

Secondly, other than our previous work using the 2017 data, the only other study of support for the SDIL focused particularly on parents.[33] This found that 1-3 months after implementation 57% of parents supported what the SDIL was trying to achieve 1-3 months after implementation. We found that those with dependent children were less likely to support the SDIL than those without (overall adjusted predicted support was 70% (95% CI: 69 to 72) in those without children, and 66% (95% CI: 64 to 68) in those with children) meaning that our sample including both those with and without dependent children would likely have higher support than one focused exclusively on parents.

Finally, previous research has found that support for SSB taxes consistently increases when it is made clear that revenues will be used for health promotion.[9 18 19 26 29 30] We indicated that the government's stated intention for SDIL revenues was to spend them "on breakfast clubs, and sports in primary schools". This may have increased support compared to others studies.

Whilst perceived effectiveness also remained high throughout, it decreased from 72% in 2017 to 67% in 2018 and 2019. We proposed two potential explanations. Firstly, it is possible that the initial decrease in perceived effectiveness reflects an assumption that the levy achieves its effects via price increases, coupled with limited experience of price increases. The Government's stated (and achieved) aim of the SDIL was to prompt reformulation and we were careful to state this in the survey.[36] Despite this, many

For peer review only - http://bmjopen.bmj.com/site/about/guidelines.xhtml

people may find it difficult to dissociate the concept of 'tax' from price increases and so assume that this is how the levy achieves its effects. This may have been reinforced by temporary signage in stores in spring 2018 explaining that any recent price increases were due to the levy.[42] The true effect of the SDIL on SSB prices was not straightforward with some taxed categories increasing in price and others decreasing.[36] Further, only 44% of parents reported noticing an increase in SSB prices following SDIL implementation.[33] Nevertheless, if participants believed the levy would only work if it increased prices and they did not consistently experience price increases, they could well conclude it was less effective than they would have predicted prior to implementation. Secondly, the drop in perceived effectiveness between 2017 (pre-implementation) and 2018 (post-implementation) may reflect the difference between a hypothetical and implemented tax, and that despite similar wording, the measures of perceived effectiveness used in these years were not entirely comparable.

The associations between socio-demographic and psychological covariates and both support for and perceived effectiveness of the SDIL largely reflect those reported in our previous analysis.[32] As these associations were not the focus of the present work, we refer readers there for a fuller consideration of the interpretation of these associations. In brief, the patterns found largely reflect an intuitive association between more 'public health' orientated attitudes and beliefs and acceptability of the SDIL.

Implications of findings

Many structural public health interventions, such as SSB taxes, require government action. This means that political support is an important determinant of implementation of such interventions. Public acceptability may be one important influence on political support. Public acceptability may not just impact on short term effectiveness via mechanisms such as cross-border shopping, but also on tax longevity and hence long-term effectiveness. Even when written into legislation such interventions are not necessarily immutable. For example, the SSB tax in Chicago, IL was repealed two months after implementation,[6] and a tax on high fat products in Denmark was repealed after a year.[7] In the UK, a proposed tax increase on hot baked goods (the 'pasty tax') was abandoned before implementation following a public outcry.[8] Given this history of repeal of structural interventions, public acceptability is likely to be an important determinant not just of initial implementation but of ongoing longevity and hence long-term impact. That public support for, and perceived effectiveness of, the SDIL remains high even after implementation may help it persist and give confidence to policymakers elsewhere that SSB taxes, and other structural public health interventions, can have high and ongoing public acceptability.

For peer review only - http://bmjopen.bmj.com/site/about/guidelines.xhtml

This may be particularly important with the recent move in the UK towards more structural policies to address obesity in the last five years.[43]

Given our finding of a small drop in perceived effectiveness between before and after implementation of the SDIL, it may be valuable to continue to monitor this. Greater understanding of what makes effective structural public health interventions more and less attractive to the public, and how they can be framed to increased acceptability is also required.

CONCLUSIONS

There is high support for the SDIL amongst UK adults and this did not change between four months before implementation and 20 months after. Perceived effectiveness of the SDIL also remained high, but there was evidence of a small decrease after implementation in 2018. This may relate to reported complexities in the impact of the SDIL on SSB prices and the difference between reporting on perceived effectiveness of a hypothetical versus implemented policy. Whilst public acceptability of structural public health interventions is recognised as an important determinant of implementation, it may also be an important determinant of policy persistence. Greater understanding of influences on public acceptability of structural public health interventions such as SSB taxes, and how it can be increased, is required.

For peer review only

FIGURE LEGEND

Figure 1. Predicted percentage (95% confidence intervals) of participants who supported (left) and perceived the Soft Drinks Industry Levy to be effective (right); International Food Policy Study, 2017-19

FUNDING

Funding for this project was provided by a Canadian Institutes of Health Research (CIHR) Project Grant (PJT-162167). Additional support was provided by an International Health Grant from the Public Health Agency of Canada (PHAC), and a PHAC - CIHR Chair in Applied Public Health (no grant number available). Collection of 2017 and 2018 UK data collection was supported by the Health Foundation (no grant numbers available). JA & MW are supported by the Medical Research Council (grant number MC_UU_ 00006/7) and the Centre for Diet and Activity Research (CEDAR), a UKCRC Public Health Research Centre of Excellence (grant number MR/K023187/1): funding for CEDAR from the British Heart Foundation, Cancer Research UK, Economic and Social Research Council, Medical Research Council, the National Institute for Health Research, and the Wellcome Trust, under the auspices of the UK Clinical Research Collaboration, is gratefully acknowledged. Views expressed in this paper are those of the authors and not necessarily those of the above named funders. The study has no affiliations with the food industry.

COMPETING INTERESTS

None declared

AUTHOR CONTRIBUTIONS

JA, DP & MW conceived the idea for this paper. JA analysed the data and drafted the manuscript. JA, TP, MW, DH and LV read and provided critical comments on the manuscript and approved the final version. DH conceived the idea for the IFPS and secured funding. DH and LV developed the first draft of survey. TP led the further development of the UK survey instrument, with input from JA, MW, DH and LV.

DATA SHARING

Data is available directly from the International Food Policy Study team on reasonable request (see www.foodpolicystudy.com).

For peer review only

REFERENCES

1. World Health Organization. Tackling NCDs: "Best Buys" and Other Recommended Interventions for the Prevention and Control of Noncommunicable Diseases. Geneva, Switzerland: WHO, 2017.

2. Teng AM, Jones AC, Mizdrak A, Signal L, Genç M, Wilson N. Impact of sugar-sweetened beverage taxes on purchases and dietary intake: Systematic review and meta-analysis. Obesity Reviews 2019;**20**:1187-204.

3. Allcott H, Lockwood BB, Taubinsky D. Should We Tax Sugar-Sweetened Beverages? An Overview of Theory and Evidence. Journal of Economic Perspectives 2019;**33**:202-27.

4. White M, Adams J. Different scientific approaches are needed to generate stronger evidence for population health improvement. PLOS Medicine 2018;**15**:e1002639.

5. Le Bodo Y, Paquette M-C, De Wals P. Social and Political Acceptability of Sugar-Sweetened Beverage Taxation. Taxing Soda for Public Health: A Canadian Perspective. Cham: Springer International Publishing, 2016:193-212.

6. Powell LM, Leider J. Evaluation of Changes in Beverage Prices and Volume Sold Following the Implementation and Repeal of a Sweetened Beverage Tax in Cook County, Illinois. JAMA Network Open 2020;**3**:e2031083-e83.

7. Stafford N. Denmark cancels "fat tax" and shelves "sugar tax" because of threat of job losses. BMJ 2012;**345**:e7889.

8. Robinson M. P-Day! Victory for pasty eaters everywhere as U-turn ends threat of 50p VAT increase (as long as you eat at Greggs or don't mind a lukewarm one) 2012. https://www.dailymail.co.uk/news/article-2151287/Pasty-tax-U-turn-ends-threat-50p-VAT-increase-long-eat-Greggs-dont-mind-lukewarm-one.html#ixzz1wN1mjr9H. Accessed 10 Feb 2021

9. Eykelenboom M, van Stralen MM, Olthof MR, et al. Political and public acceptability of a sugar-sweetened beverages tax: a mixed-method systematic review and meta-analysis. Int J Behav Nutr Phys Act 2019;**16**:78.

10. Rivard C, Smith D, McCann SE, Hyland A. Taxing sugar-sweetened beverages: a survey of knowledge, attitudes and behaviours. Public health nutrition 2012;**15**:1355-61.

11. Barry CL, Niederdeppe J, Gollust SE. Taxes on sugar-sweetened beverages: results from a 2011 national public opinion survey. American journal of preventive medicine 2013;**44**:158-63.

12. Donaldson EA, Cohen JE, Rutkow L, Villanti AC, Kanarek NF, Barry CL. Public support for a sugar-sweetened beverage tax and pro-tax messages in a Mid-Atlantic US state. Public health nutrition 2015;**18**:2263-73.

13. Jou J, Niederdeppe J, Barry CL, Gollust SE. Strategic messaging to promote taxation of sugar-sweetened beverages: lessons from recent political campaigns. American journal of public health 2014;**104**:847-53.

For peer review only

For peer review only - http://bmjopen.bmj.com/site/about/guidelines.xhtml

14. Niederdeppe J, Gollust SE, Jarlenski MP, Nathanson AM, Barry CL. News coverage of sugar-sweetened beverage taxes: pro- and antitax arguments in public discourse. American journal of public health 2013;**103**:e92-8.

15. Simon PA, Chiang C, Lightstone AS, Shih M. Public Opinion on Nutrition-Related Policies to Combat Child Obesity, Los Angeles County, 2011. Preventing Chronic Disease 2014;**11**:E96.

16. Somerville C, Marteau TM, Kinmonth AL, Cohn S. Public attitudes towards pricing policies to change health-related behaviours: a UK focus group study. European Journal of Public Health 2015;**25**:1058-64.

17. Petrescu D, Hollands G, Couturier D, Ng Y, Marteau T. Public acceptability in the UK and USA of nudging to reduce obesity: The example of reducing sugar-sweetened beverages consumption. PLoS ONE 2016;**11**:1-18.

18. Thomas-Meyer M, Mytton O, Adams J. Public responses to proposals for a tax on sugar-sweetened beverages: A thematic analysis of online reader comments posted on major UK news websites. PLOS ONE 2017;**12**:e0186750.

19. Timpson H, Lavin R, Hughes L. Exploring the Acceptability of a Tax on Sugar-Sweetened Beverages: Insight Work. Liverpool: Centre for Public Health, Liverpool John Moores University, 2013.

20. Beeken RJ, Wardle J. Public beliefs about the causes of obesity and attitudes towards policy initiatives in Great Britain. Public health nutrition 2013;**16**:2132-7.

21. Cancer Research UK, UK Health Forum. Short and sweet: why the government should introduce a sugary drinks tax in the UK. London, 2016.

22. ComRes. Daily Mail sugar tax poll 2015. http://www.comres.co.uk/polls/daily-mail-sugar-tax-poll/. Accessed 2 March 2016

23. Ipsos Mori Social Research Institute. Great Britain: The way we live now. Understanding Society 2013;**December 2013**.

24. Curry LE, Rogers T, Williams P, Homsi G, Willett J, Schmitt CL. Public Attitudes and Support for a Sugar-Sweetened Beverage Tax in America's Heartland. Health promotion practice 2017;**19**:418-26.

25. Fatemi M, Murray R, Langley T. Public acceptance of obesity prevention policies in the UK. Journal of Cancer Policy 2020;**27**:100256.

26. Cullerton K, Baker P, Adsett E, Lee A. What do the Australian public think of regulatory nutrition policies? A scoping review. Obesity Reviews 2021;**22**:e13106.

27. Kwon J, Cameron AJ, Hammond D, et al. A multi-country survey of public support for food policies to promote healthy diets: Findings from the International Food Policy Study. BMC Public Health 2019;**19**:1205.

28. Gollust SE, Barry CL, Niederdeppe J. Americans' opinions about policies to reduce consumption of sugar-sweetened beverages. Preventive Medicine 2014;**63**:52-57.

For peer review only - http://bmjopen.bmj.com/site/about/guidelines.xhtml

29. Julia C, Mejean C, Vicari F, Peneau S, Hercberg S. Public perception and characteristics related to acceptance of the sugar-sweetened beverage taxation launched in France in 2012. Public health nutrition 2015;**18**:2679-88.

30. Moretto N, Kendall E, Whitty J, et al. Yes, the government should tax soft drinks: findings from a citizens' jury in Australia. International journal of environmental research and public health 2014;**11**:2456-71.

31. Diepeveen S, Ling T, Suhrcke M, Roland M, Marteau T. Public acceptability of government intervention to change health-related behaviours: a systematic review and narrative synthesis. BMC Public Health 2013;**13**:756.

32. Pell D, Penney T, Hammond D, Vanderlee L, White M, Adams J. Support for, and perceived effectiveness of, the UK soft drinks industry levy among UK adults: cross-sectional analysis of the International Food Policy Study. BMJ Open 2019;**9**:e026698.

33. Gillison F, Grey E, Griffin T. Parents' Perceptions and Responses to the UK Soft Drinks Industry Levy. J Nutr Educ Behav 2020;**52**:626-31.

34. HM Revenue & Customs. Soft Drinks Industry Levy 2016. https://www.gov.uk/government/publications/soft-drinks-industry-levy/soft-drinks-industry-levy. Accessed 5 December

35. Gauke D. Budget 2016. London: HM Treasury, 2016.

36. Scarborough P, Adhikari V, Harrington RA, et al. Impact of the announcement and implementation of the UK Soft Drinks Industry Levy on sugar content, price, product size and number of available soft drinks in the UK, 2015-19: A controlled interrupted time series analysis. PLoS Medicine 2020;**17**:e1003025.

37. Pell D, Mytton O, Penney T, et al. Changes in British household purchases of soft drinks associated with implementation of the Soft Drinks Industry Levy: a controlled interrupted time series analysis. BMJ 2021;**372**:doi:10.1136/bmj.n254.

38. HM Government. Childhood Obesity: A Plan for Action, 2016.

39. Vanderlee L, Reid JL, White CM, et al. Evaluation of the online Beverage Frequency Questionnaire (BFQ). Nutr J 2018;**17**:73.

40. Adams J, Bateman B, Becker F, et al. Effectiveness and acceptability of parental financial incentives and quasi-mandatory schemes for increasing uptake of vaccinations in preschool children: systematic review, qualitative study and discrete choice experiment. Health technology assessment (Winchester, England) 2015;**19**:1-176.

41. Vanderlee L, White CM, Kirkpatrick SI, et al. Non-alcoholic and alcoholic beverage intakes by adults across five upper-middle and high-income countries. Journal of Nutrition 2021;**151**:140-51.

42. Smith G. More than 300 businesses hit by sugar tax - but revenues will be half expected 2018. https://www.newfoodmagazine.com/news/72139/more-than-300-sugar-tax/. Accessed 10 Feb 2021

For peer review only

For peer review only - http://bmjopen.bmj.com/site/about/guidelines.xhtml

43. Theis DRZ, White M. Is Obesity Policy in England Fit for Purpose? Analysis of Government Strategies and Policies, 1992-2020. The Milbank quarterly 2021;**99**:126-70.

For peer review only

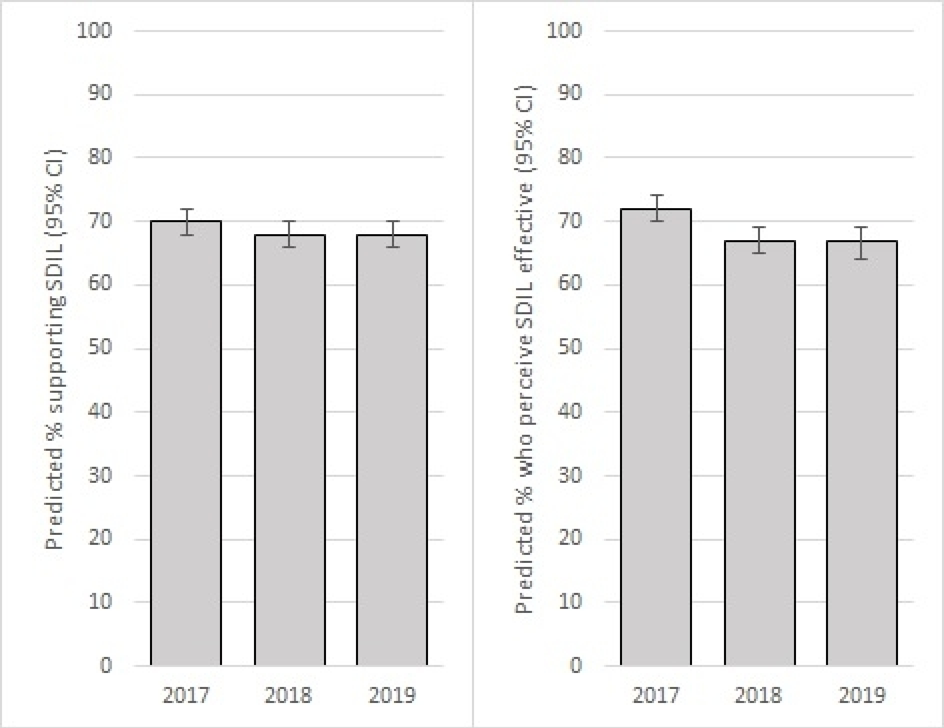

Predicted percentage (95% confidence intervals) of participants who supported (left) and perceived the Soft Drinks Industry Levy to be effective (right); International Food Policy Study, 2017-19

124x96mm (96 x 96 DPI)

STROBE Statement—Checklist of items that should be included in reports of ***cross-sectional studies***

	Item No	Recommendation	Page No
**Title and abstract**	1	(*a*) Indicate the study's design with a commonly used term in the title or the abstract	1
		(*b*) Provide in the abstract an informative and balanced summary of what was done and what was found	2
**Introduction**			
Background/rationale	2	Explain the scientific background and rationale for the investigation being reported	4-5
Objectives	3	State specific objectives, including any prespecified hypotheses	5
**Methods**			
Study design	4	Present key elements of study design early in the paper	6
Setting	5	Describe the setting, locations, and relevant dates, including periods of recruitment, exposure, follow-up, and data collection	6
Participants	6	(*a*) Give the eligibility criteria, and the sources and methods of selection of participants	6
Variables	7	Clearly define all outcomes, exposures, predictors, potential confounders, and effect modifiers. Give diagnostic criteria, if applicable	6-8
Data sources/ measurement	8*	For each variable of interest, give sources of data and details of methods of assessment (measurement). Describe comparability of assessment methods if there is more than one group	6-8
Bias	9	Describe any efforts to address potential sources of bias	6-9
Study size	10	Explain how the study size was arrived at	6
Quantitative variables	11	Explain how quantitative variables were handled in the analyses. If applicable, describe which groupings were chosen and why	6-9
Statistical methods	12	(*a*) Describe all statistical methods, including those used to control for confounding	9
		(*b*) Describe any methods used to examine subgroups and interactions	NA
		(*c*) Explain how missing data were addressed	7
		(*d*) If applicable, describe analytical methods taking account of sampling strategy	9
		(*e*) Describe any sensitivity analyses	NA
**Results**			
Participants	13*	(a) Report numbers of individuals at each stage of study—eg numbers potentially eligible, examined for eligibility, confirmed eligible, included in the study, completing follow-up, and analysed	9
		(b) Give reasons for non-participation at each stage	9
		(c) Consider use of a flow diagram	NA
Descriptive data	14*	(a) Give characteristics of study participants (eg demographic, clinical, social) and information on exposures and potential confounders	10
		(b) Indicate number of participants with missing data for each variable of interest	10
Outcome data	15*	Report numbers of outcome events or summary measures	10
Main results	16	(*a*) Give unadjusted estimates and, if applicable, confounder-adjusted estimates and their precision (eg, 95% confidence interval). Make clear which confounders were adjusted for and why they were included	12

For peer review only - http://bmjopen.bmj.com/site/about/guidelines.xhtml

For peer review only

		(*b*) Report category boundaries when continuous variables were categorized	7
		(*c*) If relevant, consider translating estimates of relative risk into absolute risk for a meaningful time period	NA
Other analyses	17	Report other analyses done—eg analyses of subgroups and interactions, and sensitivity analyses	NA
**Discussion**			
Key results	18	Summarise key results with reference to study objectives	13
Limitations	19	Discuss limitations of the study, taking into account sources of potential bias or imprecision. Discuss both direction and magnitude of any potential bias	13-14
Interpretation	20	Give a cautious overall interpretation of results considering objectives, limitations, multiplicity of analyses, results from similar studies, and other relevant evidence	14-16
Generalisability	21	Discuss the generalisability (external validity) of the study results	13
**Other information**			
Funding	22	Give the source of funding and the role of the funders for the present study and, if applicable, for the original study on which the present article is based	15

*Give information separately for exposed and unexposed groups.

Note: An Explanation and Elaboration article discusses each checklist item and gives methodological background and published examples of transparent reporting. The STROBE checklist is best used in conjunction with this article (freely available on the Web sites of PLoS Medicine at http://www.plosmedicine.org/, Annals of Internal Medicine at http://www.annals.org/, and Epidemiology at http://www.epidem.com/). Information on the STROBE Initiative is available at www.strobe-statement.org.

For peer review only

For peer review only - http://bmjopen.bmj.com/site/about/guidelines.xhtml

PUBLIC ACCEPTABILITY OF THE UK SOFT DRINKS INDUSTRY LEVY: REPEAT CROSS-SECTIONAL ANALYSIS OF THE INTERNATIONAL FOOD POLICY STUDY (2017-2019)

Journal:	*BMJ Open*
Manuscript ID	bmjopen-2021-051677.R1
Article Type:	Original research
Date Submitted by the Author:	26-Jul-2021

Complete List of Authors:	Adams, J; University of Cambridge, MRC Epidemiology Unit<br>Pell, David; University of Cambridge, MRC Epidemiology Unit; NHS RightCare, Midlands Region<br>Penney, Tarra; York University Faculty of Health, School of Global Health<br>Hammond, D; University of Waterloo, School of Public Health<br>Vanderlee, Lana; Universite Laval, Ecole de Nutrition<br>White, Martin; University of Cambridge, MRC Epidemiology Unit

**Primary Subject Heading**:	Public health
Secondary Subject Heading:	Nutrition and metabolism
Keywords:	NUTRITION & DIETETICS, PUBLIC HEALTH, Health policy < HEALTH SERVICES ADMINISTRATION & MANAGEMENT

I, the Submitting Author has the right to grant and does grant on behalf of all authors of the Work (as defined in the below author licence), an exclusive licence and/or a non-exclusive licence for contributions from authors who are: i) UK Crown employees; ii) where BMJ has agreed a CC-BY licence shall apply, and/or iii) in accordance with the terms applicable for US Federal Government officers or employees acting as part of their official duties; on a worldwide, perpetual, irrevocable, royalty-free basis to BMJ Publishing Group Ltd ("BMJ") its licensees and where the relevant Journal is co-owned by BMJ to the co-owners of the Journal, to publish the Work in this journal and any other BMJ products and to exploit all rights, as set out in our licence.

The Submitting Author accepts and understands that any supply made under these terms is made by BMJ to the Submitting Author unless you are acting as an employee on behalf of your employer or a postgraduate student of an affiliated institution which is paying any applicable article publishing charge ("APC") for Open Access articles. Where the Submitting Author wishes to make the Work available on an Open Access basis (and intends to pay the relevant APC), the terms of reuse of such Open Access shall be governed by a Creative Commons licence – details of these licences and which Creative Commons licence will apply to this Work are set out in our licence referred to above.

Other than as permitted in any relevant BMJ Author's Self Archiving Policies, I confirm this Work has not been accepted for publication elsewhere, is not being considered for publication elsewhere and does not duplicate material already published. I confirm all authors consent to publication of this Work and authorise the granting of this licence.

For peer review only

PUBLIC ACCEPTABILITY OF THE UK SOFT DRINKS INDUSTRY LEVY: REPEAT CROSS-SECTIONAL ANALYSIS OF THE INTERNATIONAL FOOD POLICY STUDY (2017-2019)

Jean Adams,[1] David Pell,[1,2†] Tarra Penney,[3] David Hammond,[4] Lana Vanderlee,[5] Martin White[1]*

[1]Centre for Diet & Activity Research, MRC Epidemiology Unit, University of Cambridge, Cambridge, UK

[2]RightCare, Midlands Region, NHS England, Newcastle upon Tyne, UK

[3]School of Global Health, York University, Toronto, Ontario, Canada

[4]School of Public Health, University of Waterloo, Waterloo, Ontario, Canada

[5]Centre NUTRISS (Nutrition, Santé et Société), École de Nutrition, Université Laval, Quebec, Quebec, Canada

[†]DP was affiliation 1 when this work was originally conceived, but is now affiliation 2

***Corresponding author:** Jean Adams, Centre for Diet & Activity Research, MRC Epidemiology Unit, University of Cambridge School of Clinical Medicine, Box 285 Institute of Metabolic Science, Cambridge Biomedical Campus, Cambridge, UK, CB2 0QQ. Email: jma79@medschl.cam.ac.uk Tel: +44 (0) 1223 769 142

Main text word count: 3493

For peer review only

ABSTRACT

Objectives To determine whether public acceptability, in terms of both support for and perceived effectiveness of, the UK Soft Drinks Industry Levy (SDIL) changed between four months prior to, and 8 and 20 months after, implementation.

Design Repeat cross-sectional on-line survey.

Setting UK

Participants UK respondents to the International Food Policy Study aged 18-64 years who provided information on all variables of interest in November-December 2017 (4 months prior to SDIL implementation), 2018 (8 months after) or 2019 (20 months after; n=10,284).

Outcome measures Self-reported support for, and perceived effectiveness of, the SDIL.

Results The adjusted logistic regression model predicted that 70% (95% CI: 68 to 72) of participants supported the SDIL in 2017, 68% (95% CI: 67 to 70) in 2018, and 68% (95% CI: 66 to 70) in 2019. There was no evidence of a difference in support in 2018 vs 2017 (odds ratio (OR): 0.93; 95% confidence intervals (CI): 0.81 to 1.05); or in 2019 vs 2017 (OR: 0.90; 95% CI (0.78 to 1.03)). The adjusted logistic regression model predicted that 72% (95% CI: 70 to 74) of participants perceived the SDIL to be effective in 2017, 67% (95% CI: 65 to 69) in 2018, and 67% (95% CI: 64 to 69) in 2019. There was evidence that perceived effectiveness decreased a small amount in 2018 vs 2017 (OR: 0.78; 95% CI: 0.69 to 0.88). The difference in 2019 vs 2017 was similar.

Conclusions We found high support for the SDIL amongst UK adults and this did not change between 4 months before implementation and 8 or 20 months after. Whilst perceived effectiveness remained high, there was evidence that this decreased slightly after implementation in 2018, but no further in 2019. Greater understanding of influences on public acceptability of effective structural public health interventions is required.

Keywords: taxation, soda tax, public health, attitudes, nutrition & dietetics

For peer review only - http://bmjopen.bmj.com/site/about/guidelines.xhtml

ARTICLE SUMMARY

Strengths and limitations of the study

- We used three annual waves of a large, population-based survey (n=10,284).
- We were careful to present the Soft Drinks Industry Levy (SDIL) as an intervention targeted at manufacturers rather than consumers, with revenues ear-marked for health-promotion activities.
- This is a repeat cross-sectional design with measures before and after implementation of the SDIL in April 2018, but all time points were after announcement of the SDIL in March 2016
- Whilst all measures have strong face validity, we have not explored other aspects of validity or reliability of any of the measures used; in many cases it would be hard to know what the 'gold standard' measure should be.

For peer review only

INTRODUCTION

Taxes on sugar sweetened beverages (SSBs) are recommended by the World Health Organisation to reduce sugar consumption and prevent non-communicable diseases.[1] Systematic review evidence suggests that SSB taxes lead to reductions in SSB purchasing and consumption, but there is substantial heterogeneity in effect sizes[2] and tax design.[3] Better understanding of the contextual factors that influence the effectiveness of SSB taxes may enable taxes to be better tailored to context.[4] Such contextual factors may include public acceptability of SSB taxes.

By their nature, regulatory policies such as SSB taxes require political support for implementation. Political support is, in turn, likely to be influenced by public acceptability. As well as influencing implementation, public acceptability may also influence the effectiveness and longevity of SSB taxes.[5] For example, if price increases following an SSB tax are not acceptable to the public, then they may travel to buy SSBs in un-taxed areas (so called cross-border shopping).[3] Further, a number of food taxes have been repealed after implementation, in part due to perceived public backlash.[6-8] This makes it important to understand how public acceptability of SSB taxes changes after implementation.

Public acceptability of a policy reflects both public support for that policy and perceptions of how effective the policy may be.[9 10] Public support for hypothetical SSB taxes ranges from around 35-60%.[11-28] A recent systematic review reported a pooled figure for support of 42% (95% confidence interval (CI): 38 to 47).[9] Associations between support and variables such as age, sex, SSB consumption and socio-economic position are inconsistent.[11 13 16 18 24-26 28-30] However, support is consistently higher when it is clear that revenue raised will be used for health promotion activities, such as subsidies on healthy food.[9 19 20 27 30 31] There is also evidence that framing of SSB taxes, particularly how SSBs are defined and what the stated aims of taxes are, can influence public support.[32]

A recent systematic review of randomised controlled trials indicates that providing information on the effectiveness (or ineffectiveness) of government policies leads to significant changes in support for those policies.[10] Perceived effectiveness of SSB taxes has been less studied than public support.[9 18 30] However, in a systematic review, pooled estimates were that 39% (95% CI 26 to 54) of the public believe SSB taxes reduce purchases and consumption, and 40% (95% CI 29 to 54) believe that they impact on health-related outcomes.[9] A perception that SSB taxes are unlikely to be effective is a common explanation for low public support in qualitative studies.[9 17 19]

For peer review only - http://bmjopen.bmj.com/site/about/guidelines.xhtml

Most previous work on public acceptability of SSB taxes has focused on hypothetical taxes. A systematic review of support for government interventions to change health-related behaviours (that did not include any studies on SSB taxes) found that support tends to be higher for implemented, rather than hypothetical, policies.[33] Similarly, perceived effectiveness may be influenced by whether respondents are reporting on a hypothetical or implemented tax.

We are aware of four studies on public acceptability of implemented SSB taxes. One study of the French excise tax on sweetened beverages found 49% of the public supported the tax and 58% believed it would improve health.[30] An international study exploring public support for a range of dietary public health policies in 2017 included data from Mexico, where an SSB tax was implemented in 2014. Whilst support for an SSB tax was higher in Mexico than other countries (54% vs 30-49%), the same was true for many other policies studied.[28] Two studies have focused on the UK Soft Drinks Industry Levy (SDIL; described in Box 1).[34 35] Our previous population-based survey conducted after announcement, but before implementation (i.e. the 2017 data presented in the current work), of the SDIL found that 70% of UK adults supported the SDIL and 71% thought it would be effective.[34] Finally, a survey of parents of children aged 5-11 years conducted soon after SDIL implementation found that 57% supported its aims.[35] We are not aware of any study exploring change in public acceptability of SSB taxes from before to after implementation.

Our aim was to determine whether public acceptability, in terms of both support for, and perceived effectiveness of, the SDIL changed between four months prior to implementation (i.e. 20 months after announcement) and 8 and 20 months after implementation.

For peer review only - http://bmjopen.bmj.com/site/about/guidelines.xhtml

Box 1 – Key characteristics of the UK Soft Drinks Industry Levy[36-39]

- Levied on companies importing or manufacturing SSBs, not consumers
- Intentional two-year delay between announcement (March 2016) and implementation (April 2018) to give manufacturers time to adapt by developing lower sugar products
- Tiered with eligible drinks containing ≥8g of sugar per 100ml charged £0.24 [€0.27, US$0.33]/litre, those containing ≥5g but <8g charged £0.18 [€0.20, US$0.24]/litre, and those containing <5g not charged
- Exemptions for pure fruit juices, milk-based drinks and a number of other smaller categories
- Announcement included a statement that revenue raised would be spent on school sport and school breakfast clubs
- Associated with substantial reformulation of the UK soft drinks market to reduce sugar content
- Associated with complex changes in SSB prices with some categories increasing in price and others decreasing
- Associated with no change in volume of all drinks purchased, but a reduction in sugar purchased from drinks

METHODS

We used repeat cross-sectional survey data from the International Food Policy Study (IFPS). We conducted both unadjusted analyses and analyses adjusted for a number of socio-demographic and psychological variables that are potential, or previously reported, correlates of support for, and perceived effectiveness of, the SDIL.

Sampling, recruitment and data collection

Data were from UK participants in the 2017, 2018 and 2019 waves of IFPS. This is an annual repeat cross-sectional survey conducted in Australia, Canada, Mexico, the UK and the USA with an annual, pragmatic, recruitment target of 4000 adults per country per wave. Data were collected via self-completed web surveys in November-December each year, representing 4-5 months before implementation, but 19-20 months after announcement (2017); 7-8 months after implementation (2018) and 19-20 months after implementation (2019). Respondents were recruited through Nielsen Consumer Insights Global Panel and their partners' panels. Email invitations with unique survey access links were sent to a random sample of panellists within each country after targeting for demographics;

For peer review only - http://bmjopen.bmj.com/site/about/guidelines.xhtml

For peer review only

panellists known to be ineligible were not invited. Potential respondents were screened for eligibility and quota requirements based on age and sex. UK participation rates (i.e. "the number of respondents who provided a usable response divided by the total number of initial personal invitations requesting participation")[40] were 7.4%, 11.5% and 4.5% in 2017, 2018 and 2019 respectively.

Respondents provided informed consent prior to completing the survey and received remuneration in accordance with their panel's usual incentive structure (e.g. points-based or monetary rewards, or chances to win prizes). A full description of the study methods can be found at www.foodpolicystudy.com/methods.

Inclusion criteria

We included in the analysis UK resident participants in the 2017, 2018 and 2019 IFPS waves who met the following criteria: were aged 18-64 years, provided information on sex at birth and age, and passed data quality checks (provided a valid response to a data quality question; took at least 15 minutes to complete the survey; and provided a valid response to at least three of 20 open-ended measures).

Variables used in the analysis

The variables used in the analysis, the survey items from which they were derived, response options and how response options were collapsed for analysis are described in Table 1.

For peer review only

For peer review only - http://bmjopen.bmj.com/site/about/guidelines.xhtml

Table 1. Description of items and response options used in the analysis

		Response options	
**Concept**	**Item wording (where applicable)**	**All**	**Categories used in the analysis**
Age	How old are you?	In years	18-24 years
			25-34 years
			35-44 years
			45-54 years
			55-64 years
Sex	What sex were you assigned at birth, meaning on your original birth certificate?	Female	Female
		Male	Male
Education	What is the highest level of education you have completed?	Qualifications not listed below, free-text equivalents, Don't Know, Refuse to answer	School level
		NVQ Level 4-5, HNC, HND, RSA Higher Diploma, BTEC Higher Level, Degree, Higher Degree, free-text equivalents	Post school level
Income sufficiency	How easy is it to make ends meet?	Neither easy nor difficult, Difficult, Very difficult, Don't know, Refuse to answer	Not easy
		Very easy, Easy	Easy
Children	Do you have any children (including step-children or adopted children) under the age of 18?	No, Don't know, Refuse to answer	No
		Yes	Yes
SSB consumption	[Calculated from Beverage Frequency Questionnaire: reported consumption over last 7 days]	Any consumption of non-diet Fizzy drinks, Sweetened fruit juice drinks, Regular sports drinks, Regular energy drinks, or Spirits with mixers that have calories	Consumers
		No consumption of above	Non-consumers
Social norms	People important to me try not to drink sugary drinks	Neither agree nor disagree, Disagree, Strongly disagree, Don't know, Refuse to answer	Not agree
		Strongly agree, Agree	Agree
Attitudes	Sugary drinks taste good	Strongly agree, Agree	Agree
		Neither agree nor disagree, Disagree, Strongly disagree, Don't know, Refuse to answer	Not agree
Knowledge	Frequently drinking sugary drinks increases the risk of obesity	False, Don't know, Refuse to answer	Not true
		True	True
Expert trust	I trust messages from health experts on sugary drinks	Neither agree nor disagree, Disagree, Strongly disagree, Don't know, Refuse to answer	Not agree
		Strongly agree, Agree	Agree
Industry trust	I trust messages from the food and beverage industry on sugary drinks	Neither agree nor disagree, Disagree, Strongly disagree, Don't know, Refuse to answer	Not agree
		Strongly agree, Agree	Agree
Support	In 2018 a new sugary drink tax will be/was introduced in the UK. This aims to encourage manufacturers to reduce the sugar in drinks. The money will be spent on breakfast clubs, and sports in primary schools. Do you support or oppose this policy?	Strongly support, Support	Support
		Oppose, Strongly oppose, Don't know, Refuse to answer	Oppose

For peer review only - http://bmjopen.bmj.com/site/about/guidelines.xhtml
For peer review only

Effectiveness	Preamble as above. How effective do you think these kinds of policies would be/are?	Somewhat effective, Mostly effective, Very effective	Effective
		Not at all effective, Don't know, Refuse to answer	Not effective

Note. SSB = sugar sweetened beverage; NVQ = National Vocational Qualification; HNC = Higher National Certificate; HND = Higher National Diploma, RSA = Royal Society of Arts; BTEC = Business and Technology Education Council

For peer review only

Outcome variables

The outcome variables of interest were single-item measures of support for, and perceived effectiveness of, the SDIL collapsed into binary categories of support vs oppose and effective vs not-effective as described in Table 1.

Exposure variable

The exposure variable of interest was year – either 2017, 2018 or 2019.

Potential confounding variables

We adjusted for a number of individual-level socio-demographic and psychological variables in the analysis (see Table 1 for details). With the exception of age, education and income sufficiency, these all showed associations with one or both outcome variables in our previous analysis of 2017 data.[34] Given that age and markers of socio-economic position, such as education and income sufficiency, have been previously, if inconsistently, associated with public support for SSB taxes,[11 13 16 18 24-26 29 30] we included them here, despite no relationship with the outcomes in our previous analysis.

We included single-item measures of attitudes, knowledge, and social norms related to sugary drinks; and trust in advice on sugary drinks from health experts and the food and beverage industry. As previous research has indicated that the acceptability of food taxes varies with the stated intentions of these,[9 19 20 27 30 31] we included a preamble to the questions about support for, and perceived effectiveness of, the SDIL outlining the intention of the levy and the stated use of revenue generated.

Socio-demographic variables considered were age in approximately 10-year age bands, sex at birth, whether or not participants had dependent children, and socio-economic position. Parental status was included as the SDIL has been particularly framed in terms of potential benefits for children.[37 41] Socio-economic position was measured using participants' highest educational qualification and perceived income sufficiency. Income sufficiency has previously been associated with financial resources and health outcomes[42] and provides a comparable measure across the range of different economic settings in IFPS.

Current behaviour has previously been associated with perceived acceptability of public health interventions,[33] and we found that SSB consumers were less likely to support the SDIL in 2017. As such, we adjusted for SSB consumption using the Beverage Frequency Questionnaire. This is a 7-day food record that assesses consumption of 17 beverage categories, including caloric and non-caloric beverages.[43] For each beverage category, respondents report the number of drinks and the usual

For peer review only - http://bmjopen.bmj.com/site/about/guidelines.xhtml

portion size using category-specific images of beverage containers adapted from the Automated Self-Administered 24 hour (ASA24) dietary assessment tool. Participants who reported any consumption of regular fizzy drinks, alcoholic drinks with regular mixers or cocktails that have calories, sweetened fruit drinks, sports drinks, or energy drinks over the previous seven days were considered SSB consumers.

Analysis

Data were weighted with post-stratification sample weights constructed using population estimates from the UK census based on age group, sex and region. These were used throughout the analysis to reduce the effects of non-response and selection bias. We included 'don't know' and 'refuse to answer' responses as described in Table 1, meaning there was no missing data.

Descriptive statistics were used to quantify all variables of interest. Logistic regression models were fitted to explore associations between study wave and the binary measures of support for, and perceived effectiveness of, the SDIL before and after adjustment for other variables. We used separate models to explore support for the SDIL and perceived effectiveness of the SDIL. In these models support or perceived effectiveness were the outcome variables, study wave was the exposure variable, and all other variables in Table 1 were covariates. Standard errors were not clustered. We used the fully adjusted models, and mean values of covariates, to predict the proportion of the population likely to be supportive of the SDIL, and think it would be effective, at each time point.

Data were analysed using Stata version 15.

Ethics

The study received ethical clearance from a University of Waterloo Research Ethics Committee (ORE# 21460 and ORE# 30829). All participants provided informed consent to take part.

Patient and public involvement

Patients and the public were not involved in the design, conduct, analysis or interpretation of the study.

RESULTS

A total of 25,692 adults took part in IFPS across all included countries in 2017, 28,684 in 2018 and 29,290 in 2019. After removing respondents with missing data on sex at birth and age, and those who did not meet data quality checks, 18,878 (73.5%) respondents remained in 2017, 22,824 (79.6%) in 2017 and 20,968 (71.6%) in 2019. Of these, 4047 were from the UK in 2017, 5549 in 2018, and 4139 in 2019. Amongst these UK participants, 3104 (76.7%) met the additional inclusion criteria for the current work

For peer review only - http://bmjopen.bmj.com/site/about/guidelines.xhtml

(i.e. aged 18-64 years) in 2017, 4118 (74.2%) in 2018, and 3062 (74.0%) in 2019. Characteristics of the analytical sample (after applying survey weights) are described in Table 2.

For peer review only

Table 2. Weighted unadjusted characteristics of UK participants; International Food Policy Study, 2017-19

Concept	Question wording	Response category	Weighted percentage (95% confidence intervals)			
			2017, n=3104	2018, n=4118	2019, n=3062	Total, n=10,284
Age	How old are you?	18-24 years	13 (12 to 15)	12 (11 to 14)	11 (10 to 12)	12 (11 to 13)
		25-34 years	22 (20 to 23)	24 (23 to 26)	22 (20 to 24)	23 (22 to 24)
		35-44 years	20 (18 to 22)	21 (20 to 23)	23 (22 to 25)	21 (21 to 22)
		45-54 years	24 (22 to 26)	22 (20 to 23)	20 (19 to 22)	22 (21 to 23)
		55-64 years	21 (19 to 23)	21 (19 to 22)	23 (22 to 25)	22 (21 to 22)
Sex	What sex were you assigned at birth, meaning on your original birth certificate?	Female	48 (46 to 50)	50 (48 to 52)	50 (48 to 52)	49 (48 to 51)
Education	What is the highest level of education you have completed?	School level	61 (59 to 63)	74 (72 to 75)	74 (73 to 76)	70 (69 to 71)
Income sufficiency	How easy is it to make ends meet?	Not easy	61 (59 to 63)	66 (65 to 68)	66 (64 to 68)	65 (64 to 66)
Children	Do you have any children (including step-children or adopted) under 18?	No	63 (61 to 65)	68 (66 to 70)	65 (62 to 67)	66 (64 to 67)
SSB consumption	Consumed regular fizzy drinks, sweetened fruit drinks, sports drinks, energy drinks in last week	Consumers	53 (50 to 55)	44 (42 to 45)	44 (42 to 47)	46 (45 to 48)
Social norms	People important to me try not to drink sugary drinks	Not agree	46 (44 to 48)	52 (50 to 54)	51 (48 to 53)	50 (48 to 51)
Attitudes	Sugary drinks taste good	Agree	62 (60 to 64)	64 (63 to 66)	59 (57 to 61)	62 (61 to 63)
Knowledge	Frequently drinking sugary drinks increases the risk of obesity	Not true	10 (9 to 12)	14 (13 to 16)	12 (10 to 13)	12 (12 to 13)
Expert trust	I trust messages from health experts on sugary drinks	Not agree	39 (37 to 41)	40 (38 to 42)	41 (39 to 43)	40 (39 to 41)
Industry trust	I trust messages from the food and beverage industry on sugary drinks	Not agree	73 (71 to 75)	69 (67 to 70)	68 (66 to 70)	70 (69 to 71)
Support	In 2018 a new sugary drink tax will be/was introduced in the UK. This aims to encourage manufacturers to reduce the sugar in drinks. The money will be spent on breakfast clubs, and sports in primary schools. Do you support or oppose this policy?	Support	70 (68 to 72)	66 (64 to 68)	66 (64 to 68)	67 (66 to 68)
Effectiveness	Preamble as above. How effective do you think these kinds of policies are?	Effective	71 (69 to 73)	66 (64 to 67)	65 (63 to 67)	67 (66 to 68)

Note. SSB = sugar sweetened beverage

For peer review only - http://bmjopen.bmj.com/site/about/guidelines.xhtml

For peer review only

Table 3 shows the results of logistic regression analyses of associations between survey wave and support for, and perceived effectiveness of, the SDIL, before and after adjusting for the socio-demographic and psychological concepts listed. In unadjusted analyses, both support for, and perceived effectiveness of, the SDIL dropped between 2017 and 2018, but there was little difference in effect estimates in 2018 vs 2017 and in 2019 vs 2017.

In adjusted analyses there was no evidence that the proportion of participants supporting the SDIL changed between 2017 and either 2018 or 2019. In contrast, the proportion who perceived the SDIL to be effective in 2018 and 2019 was lower than that in 2017. However, the difference in the proportion who perceived the SDIL to be effective was very similar in 2018 vs 2017 and 2019 vs 2017 indicating that the decreased in perceived effectiveness occurred between 2017 and 2018.

Holding all other variables at their mean levels, the adjusted logistic regression model predicted that 70% (95% CI: 68 to 72) of participants supported the SDIL in 2017, 68% (95% CI: 67 to 70%) in 2018, and 68% (95% CI: 66 to 70) in 2019 (Figure 1). Comparable figures for perceived effectiveness were 72% (95% CI: 70 to 74) in 2017, 67% (95% CI: 65 to 69) in 2018, and 67% (95% CI: 64 to 69) in 2019 (Figure 1).

Other variables in the adjusted models were also associated with support for, and perceived effectiveness of, the SDIL (Table 3). Greater support for the SDIL was associated with: having a higher level of education, not having children at home, being a non-consumer of SSBs, having social norms to avoid sugary drinks, disliking the taste of sugary drinks, recognising an association between sugary drinks and obesity, trusting health expert messages on sugary drinks and not trusting industry messages on sugary drinks. Individuals aged 35-64 years were also more likely to support the SDIL than those aged 18-24 years, with some evidence of a step wise increase in likelihood of support across successive age groups. Greater perceived effectiveness of the SDIL was associated with: having children at home, having social norms to avoid sugary drinks, disliking the taste of sugary drinks, trusting health expert messages on sugary drinks and trusting industry messages on sugary drinks. Individuals aged 25-64 years were also less likely to perceive the SDIL to be effective. There was a stepwise decrease in perceived effectiveness across successive age groups to age 54 years, but not thereafter.

For peer review only - http://bmjopen.bmj.com/site/about/guidelines.xhtml

Table 3. Adjusted odds ratios (95% confidence intervals) of the association between year and support for, and perceived effectiveness of, the Soft Drinks Industry Levy; International Food Policy Study, 2017-19

Concept	Question wording (where applicable)	Response category	Support for the SDIL		Perceived effectiveness of the SDIL	
			Unadjusted	Adjusted	Unadjusted	Adjusted
Survey wave	Not applicable	2017	Reference	Reference	Reference	Reference
		2018	**0.84 (0.75 to 0.95)**	0.93 (0.81 to 1.05)	**0.77 (0.68 to 0.87)**	**0.78 (0.69 to 0.88)**
		2019	**0.84 (0.74 to 0.96)**	0.90 (0.78 to 1.03)	**0.76 (0.67 to 0.87)**	**0.76 (0.66 to 0.86)**
Age	How old are you?	18-24		Reference		Reference
		25-34		1.07 (0.89 to 1.28)		**0.75 (0.61 to 0.91)**
		35-44		**1.28 (1.06 to 1.55)**		**0.50 (0.41 to 0.62)**
		45-54		**1.57 (1.30 to 1.90)**		**0.46 (0.37 to 0.56)**
		55-64		**1.81 (1.50 to 2.19)**		**0.47 (0.39 to 0.58)**
Sex	What sex were you assigned at birth, meaning on your original birth certificate?	Female		Reference		Reference
		Male		1.01 (0.92 to 1.13)		1.05 (0.95 to 1.16)
Education	What is the highest level of education you have completed?	School level		Reference		Reference
		Post school level		**1.19 (1.07 to 1.32)**		1.02 (0.92 to 1.13)
Income sufficiency	How easy is it to make ends meet?	Not easy		Reference		Reference
		Easy		1.07 (0.96 to 1.20)		1.02 (0.92 to 1.14)
Dependent children	Do you have any children (including step-children or adopted children) under 18?	No		Reference		Reference
		Yes		**0.82 (0.73 to 0.92)**		**1.21 (1.07 to 1.36)**
SSB consumption	Consumed regular fizzy drinks, sweetened fruit drinks, sports drinks, energy drinks in last week	Consumers		Reference		Reference
		Non-consumers		**1.12 (1.01 to 1.25)**		1.01 (0.91 to 1.12)
Social norms	People important to me try not to drink sugary drinks	Not agree		Reference		Reference
		Agree		**1.35 (1.21 to 1.50)**		**1.35 (1.21 to 1.50)**
Attitudes	Sugary drinks taste good	Agree		Reference		Reference
		Not agree		**1.32 (1.21 to 1.50)**		**1.33 (1.19 to 1.48)**
Knowledge	Frequently drinking sugary drinks increases the risk of obesity	Not true		Reference		Reference
		True		**2.78 (2.37 to 3.27)**		**1.26 (1.07 to 1.48)**
Expert trust	I trust messages from health experts on	Not agree		Reference		Reference

For peer review only - http://bmjopen.bmj.com/site/about/guidelines.xhtml

	sugary drinks	Agree	**2.36 (2.09 to 2.66)**	**1.96 (1.75 to 2.19)**
Industry trust	I trust messages from the food and beverage industry on sugary drinks	Not agree	Reference	Reference
		Agree	**0.72 (0.63 to 0.82)**	**1.53 (1.34 to 1.74)**

Note. SDIL = Soft Drinks Industry Levy; **BOLD** indicates statistically significant at the p<0.05 level; adjusted for all concepts listed

For peer review only

DISCUSSION

Summary of findings

As far as we are aware, this is the first study to explore whether public acceptability of an SSB tax, operationalised in terms of support and perceived effectiveness, changed from before to after implementation of the tax. It also adds to the small existing literature on public acceptability of implemented (rather than hypothetical) SSB taxes.[28 30 34 35] In this population-based, repeat cross-sectional survey, after adjustment for a range of socio-demographic and psychological covariates, we found that predicted support for the SDIL remained consistently high throughout (68-70%), with no evidence that support changed from four months before to 20 months after implementation. Whilst perceived effectiveness of the SDIL was also high throughout (67-72%), there was evidence that predicted perceived effectiveness of the SDIL decreased from 72% before implementation to 67% after implementation. This change was evident 7-8 months after implementation, with no further decrease 12 months later.

Strengths and weaknesses of methods

Key strengths of the analysis are the large (relatively to other work in the field)[9], population-based, sample; inclusion of a range of socio-demographic, consumption and psychological variables; the context of an implemented, rather than hypothetical, SSB tax in the latter two time points; and consistency of methods across all three time points. Given previous findings showing that support for SSB taxes is greater when revenues are used for health-promoting activities,[9 19 20 27 30 31] we were careful to present the SDIL with revenues ear-marked for health-promotion activities. We also clearly stated that it was an intervention designed to target manufacturers rather than consumers. Social desirability bias may also be less likely to occur in more anonymous settings such as on-line surveys.[44]

Participants were recruited using non-probability sampling. Despite the use of weights for age, sex and region, the findings do not necessarily provide nationally representative estimates; and are limited to ages 18-64 years. Whilst the pattern of results in terms of patterns of associations between variables are likely to be generalizable to the UK, the estimates of absolute frequency may not be. Given international differences in dietary public health policy,[45] the pattern of findings may not be generalizable beyond the UK. Although the IFPS takes place in a number of countries key questions used here were only asked of UK participants. All data were collected after announcement of the SDIL in March 2016.

For peer review only - http://bmjopen.bmj.com/site/about/guidelines.xhtml

All variables were self-reported. Whilst all have strong face validity and the Beverage Frequency Questionnaire performs well compared to a seven day food record,[43] we have not explored validity or reliability of the other measures used. However, many were derived from existing instruments. Further, we were reliant on data availability and did not have consistent information across all three included waves on additional variables that may of relevance including: household income, age of children in the household and personality traits such as extraversion, agreeableness, conscientiousness, neuroticism or openness to experience. Although adults aged 65 years and older were included in IFPS from 2018 onwards, they were not in 2017.

Comparison to previous results and interpretation of findings

Overall, there was high support for the SDIL throughout with no evidence of significant change across years. The 68-70% prevalence of support we found is noticeably higher than the previously reported ranges of 35-60%[11-27] and a pooled estimate of 42%.[9] We propose three potential reasons why support here may be higher than previously reported. Firstly, most previous data on support for SSB taxes has been collected in the context of hypothetical taxes. Previous systematic review evidence suggests that support for governmental public health interventions tends to increase after implementation.[33] Whilst 2017 data was collected four months prior to implementation of the SDIL, the policy intention was announced in March 2016, 20 months before 2017 data collection. As such, many participants may have either believed the SDIL had already been implemented, or at least accepted that it was going to be implemented, at the time of 2017 data collection. Comparable pre-announcement data is not available.

Secondly, other than our previous work using the 2017 data, the only other study of support for the SDIL focused particularly on parents.[35] This found that 1-3 months after implementation 57% of parents supported what the SDIL was trying to achieve 1-3 months after implementation. We found that those with dependent children were less likely to support the SDIL than those without (overall adjusted predicted support was 70% (95% CI: 69 to 72) in those without children, and 66% (95% CI: 64 to 68) in those with children) meaning that our sample including both those with and without dependent children would likely have higher support than one focused exclusively on parents.

Finally, previous research has found that support for SSB taxes consistently increases when it is made clear that revenues will be used for health promotion.[9 19 20 27 30 31] We indicated that the government’s stated intention for SDIL revenues was to spend them “on breakfast clubs, and sports in primary schools”. This may have increased support compared to others studies.

For peer review only - http://bmjopen.bmj.com/site/about/guidelines.xhtml

Whilst perceived effectiveness also remained high throughout, it decreased from 72% in 2017 to 67% in 2018 and 2019. We propose three potential explanations. Firstly, it is possible that the initial decrease in perceived effectiveness reflects an assumption that the levy achieves its effects via price increases, coupled with limited experience of price increases. The Government's stated (and achieved) aim of the SDIL was to prompt reformulation and we were careful to state this in the survey.[38] Despite this, many people may find it difficult to dissociate the concept of 'tax' from price increases and so assume that this is how the levy achieves its effects. This may have been reinforced by temporary signage in stores in spring 2018 explaining that any recent price increases were due to the levy.[46] The true effect of the SDIL on SSB prices was not straightforward with some taxed categories increasing in price and others decreasing.[38] Further, only 44% of parents reported noticing an increase in SSB prices following SDIL implementation.[35] Nevertheless, if participants believed the levy would only work if it increased prices and they did not consistently experience price increases, they could well conclude it was less effective than they would have predicted prior to implementation. Secondly, the drop in perceived effectiveness between 2017 (pre-implementation) and 2018 (post-implementation) may reflect the difference between a hypothetical and implemented tax, and that despite similar wording, the measures of perceived effectiveness used in these years were not entirely comparable. Finally, media framing of the SDIL may have changed over time, influencing perceptions of its effectiveness. Although there have been analyses of media coverage of the announcement of the SDIL, we are not aware of any work that has tracked this longitudinally.[47 48]

The associations between socio-demographic and psychological covariates and both support for and perceived effectiveness of the SDIL largely reflect those reported in our previous analysis.[34] As these associations were not the focus of the present work, we refer readers there for a fuller consideration of the interpretation of these associations. In brief, the patterns found largely reflect an intuitive association between more 'public health' orientated attitudes and beliefs and acceptability of the SDIL.

Implications of findings

Many structural public health interventions, such as SSB taxes, require government action. This means that political support is an important determinant of implementation of such interventions. Public acceptability may be one important influence on political support. Public acceptability may not just impact on short term effectiveness via mechanisms such as cross-border shopping, but also on tax longevity and hence long-term effectiveness. Even when written into legislation such interventions are not necessarily immutable. For example, the SSB tax in Chicago, IL was repealed two months after

For peer review only - http://bmjopen.bmj.com/site/about/guidelines.xhtml

implementation,[6] and a tax on high fat products in Denmark was repealed after a year.[7] In the UK, a proposed tax increase on hot baked goods (the 'pasty tax') was abandoned before implementation following a public outcry.[8] Given this history of repeal of structural interventions, public acceptability is likely to be an important determinant not just of initial implementation but of ongoing longevity and hence long-term impact. That public support for, and perceived effectiveness of, the SDIL remains high even after implementation may help it persist and give confidence to policymakers elsewhere that SSB taxes, and other structural public health interventions, can have high and ongoing public acceptability. This may be particularly important with the recent move in the UK towards more structural policies to address obesity in the last five years.[49]

Given our finding of a small drop in perceived effectiveness between before and after implementation of the SDIL, it may be valuable to continue to monitor this. Greater understanding of what makes effective structural public health interventions more and less attractive to the public, and how they can be framed to increased acceptability is also required.

CONCLUSIONS

We found high levels of support for the SDIL amongst UK adults and no evidence that this changed between four months before implementation and 20 months after. We also found that perceived effectiveness of the SDIL remained high, but there was evidence of a small decrease after implementation in 2018. This may relate to reported complexities in the impact of the SDIL on SSB prices and the difference between reporting on perceived effectiveness of a hypothetical versus implemented policy. Whilst public acceptability of structural public health interventions is recognised as an important determinant of implementation, it may also be an important determinant of policy persistence. Greater understanding of influences on public acceptability of structural public health interventions such as SSB taxes, and how it can be increased, is required.

For peer review only - http://bmjopen.bmj.com/site/about/guidelines.xhtml

FIGURE LEGEND

Figure 1. Predicted percentage (95% confidence intervals) of participants who supported (left) and perceived the Soft Drinks Industry Levy to be effective (right); International Food Policy Study, 2017-19

FUNDING

Funding for this project was provided by a Canadian Institutes of Health Research (CIHR) Project Grant (PJT-162167). Additional support was provided by an International Health Grant from the Public Health Agency of Canada (PHAC, no grant number available), and a PHAC - CIHR Chair in Applied Public Health (no grant number available). Collection of 2017 and 2018 UK data collection was supported by the Health Foundation (no grant numbers available). JA & MW are supported by the Medical Research Council (grant number MC_UU_ 00006/7) and the Centre for Diet and Activity Research (CEDAR) which is a UKCRC Public Health Research Centre of Excellence (grant number MR/K023187/1): funding for CEDAR from the British Heart Foundation, Cancer Research UK, Economic and Social Research Council, Medical Research Council, the National Institute for Health Research, and the Wellcome Trust, under the auspices of the UK Clinical Research Collaboration, is gratefully acknowledged. Views expressed in this paper are those of the authors and not necessarily those of the above named funders. The study has no affiliations with the food industry.

COMPETING INTERESTS

None declared

AUTHOR CONTRIBUTIONS

JA, DP & MW conceived the idea for this paper. JA analysed the data and drafted the manuscript. JA, TP, MW, DH and LV read and provided critical comments on the manuscript and approved the final version. DH conceived the idea for the IFPS and secured funding. DH and LV developed the first draft of survey. TP led the further development of the UK survey instrument, with input from JA, MW, DH and LV.

DATA SHARING

Data is available directly from the International Food Policy Study team on reasonable request (see www.foodpolicystudy.com).

For peer review only

For peer review only - http://bmjopen.bmj.com/site/about/guidelines.xhtml

REFERENCES

1. World Health Organization. Tackling NCDs: "Best Buys" and Other Recommended Interventions for the Prevention and Control of Noncommunicable Diseases. Geneva, Switzerland: WHO, 2017.

2. Teng AM, Jones AC, Mizdrak A, Signal L, Genç M, Wilson N. Impact of sugar-sweetened beverage taxes on purchases and dietary intake: Systematic review and meta-analysis. Obesity Reviews 2019;**20**:1187-204.

3. Allcott H, Lockwood BB, Taubinsky D. Should We Tax Sugar-Sweetened Beverages? An Overview of Theory and Evidence. Journal of Economic Perspectives 2019;**33**:202-27.

4. White M, Adams J. Different scientific approaches are needed to generate stronger evidence for population health improvement. PLOS Medicine 2018;**15**:e1002639.

5. Le Bodo Y, Paquette M-C, De Wals P. Social and Political Acceptability of Sugar-Sweetened Beverage Taxation. Taxing Soda for Public Health: A Canadian Perspective. Cham: Springer International Publishing, 2016:193-212.

6. Powell LM, Leider J. Evaluation of Changes in Beverage Prices and Volume Sold Following the Implementation and Repeal of a Sweetened Beverage Tax in Cook County, Illinois. JAMA Network Open 2020;**3**:e2031083-e83.

7. Stafford N. Denmark cancels "fat tax" and shelves "sugar tax" because of threat of job losses. BMJ 2012;**345**:e7889.

8. Robinson M. P-Day! Victory for pasty eaters everywhere as U-turn ends threat of 50p VAT increase (as long as you eat at Greggs or don't mind a lukewarm one) 2012. https://www.dailymail.co.uk/news/article-2151287/Pasty-tax-U-turn-ends-threat-50p-VAT-increase-long-eat-Greggs-dont-mind-lukewarm-one.html#ixzz1wN1mjr9H. Accessed 10 Feb 2021

9. Eykelenboom M, van Stralen MM, Olthof MR, et al. Political and public acceptability of a sugar-sweetened beverages tax: a mixed-method systematic review and meta-analysis. Int J Behav Nutr Phys Act 2019;**16**:78.

10. Reynolds JP, Stautz K, Pilling M, van der Linden S, Marteau TM. Communicating the effectiveness and ineffectiveness of government policies and their impact on public support: a systematic review with meta-analysis. Royal Society Open Science 2020;**7**:190522.

11. Rivard C, Smith D, McCann SE, Hyland A. Taxing sugar-sweetened beverages: a survey of knowledge, attitudes and behaviours. Public health nutrition 2012;**15**:1355-61.

12. Barry CL, Niederdeppe J, Gollust SE. Taxes on sugar-sweetened beverages: results from a 2011 national public opinion survey. American journal of preventive medicine 2013;**44**:158-63.

13. Donaldson EA, Cohen JE, Rutkow L, Villanti AC, Kanarek NF, Barry CL. Public support for a sugar-sweetened beverage tax and pro-tax messages in a Mid-Atlantic US state. Public health nutrition 2015;**18**:2263-73.

For peer review only - http://bmjopen.bmj.com/site/about/guidelines.xhtml

14. Jou J, Niederdeppe J, Barry CL, Gollust SE. Strategic messaging to promote taxation of sugar-sweetened beverages: lessons from recent political campaigns. American journal of public health 2014;**104**:847-53.

15. Niederdeppe J, Gollust SE, Jarlenski MP, Nathanson AM, Barry CL. News coverage of sugar-sweetened beverage taxes: pro- and antitax arguments in public discourse. American journal of public health 2013;**103**:e92-8.

16. Simon PA, Chiang C, Lightstone AS, Shih M. Public Opinion on Nutrition-Related Policies to Combat Child Obesity, Los Angeles County, 2011. Preventing Chronic Disease 2014;**11**:E96.

17. Somerville C, Marteau TM, Kinmonth AL, Cohn S. Public attitudes towards pricing policies to change health-related behaviours: a UK focus group study. European Journal of Public Health 2015;**25**:1058-64.

18. Petrescu D, Hollands G, Couturier D, Ng Y, Marteau T. Public acceptability in the UK and USA of nudging to reduce obesity: The example of reducing sugar-sweetened beverages consumption. PLoS ONE 2016;**11**:1-18.

19. Thomas-Meyer M, Mytton O, Adams J. Public responses to proposals for a tax on sugar-sweetened beverages: A thematic analysis of online reader comments posted on major UK news websites. PLOS ONE 2017;**12**:e0186750.

20. Timpson H, Lavin R, Hughes L. Exploring the Acceptability of a Tax on Sugar-Sweetened Beverages: Insight Work. Liverpool: Centre for Public Health, Liverpool John Moores University, 2013.

21. Beeken RJ, Wardle J. Public beliefs about the causes of obesity and attitudes towards policy initiatives in Great Britain. Public health nutrition 2013;**16**:2132-7.

22. Cancer Research UK, UK Health Forum. Short and sweet: why the government should introduce a sugary drinks tax in the UK. London, 2016.

23. ComRes. Daily Mail sugar tax poll 2015. http://www.comres.co.uk/polls/daily-mail-sugar-tax-poll/. Accessed 2 March 2016

24. Ipsos Mori Social Research Institute. Great Britain: The way we live now. Understanding Society 2013;**December 2013**.

25. Curry LE, Rogers T, Williams P, Homsi G, Willett J, Schmitt CL. Public Attitudes and Support for a Sugar-Sweetened Beverage Tax in America's Heartland. Health promotion practice 2017;**19**:418-26.

26. Fatemi M, Murray R, Langley T. Public acceptance of obesity prevention policies in the UK. Journal of Cancer Policy 2020;**27**:100256.

27. Cullerton K, Baker P, Adsett E, Lee A. What do the Australian public think of regulatory nutrition policies? A scoping review. Obesity Reviews 2021;**22**:e13106.

28. Kwon J, Cameron AJ, Hammond D, et al. A multi-country survey of public support for food policies to promote healthy diets: Findings from the International Food Policy Study. BMC Public Health 2019;**19**:1205.

For peer review only

For peer review only - http://bmjopen.bmj.com/site/about/guidelines.xhtml

29. Gollust SE, Barry CL, Niederdeppe J. Americans' opinions about policies to reduce consumption of sugar-sweetened beverages. Preventive Medicine 2014;**63**:52-57.

30. Julia C, Mejean C, Vicari F, Peneau S, Hercberg S. Public perception and characteristics related to acceptance of the sugar-sweetened beverage taxation launched in France in 2012. Public health nutrition 2015;**18**:2679-88.

31. Moretto N, Kendall E, Whitty J, et al. Yes, the government should tax soft drinks: findings from a citizens' jury in Australia. International journal of environmental research and public health 2014;**11**:2456-71.

32. Rowbotham S, McKinnon M, Marks L, Hawe P. Research on media framing of public policies to prevent chronic disease: A narrative synthesis. Social Science & Medicine 2019;**237**:112428.

33. Diepeveen S, Ling T, Suhrcke M, Roland M, Marteau T. Public acceptability of government intervention to change health-related behaviours: a systematic review and narrative synthesis. BMC Public Health 2013;**13**:756.

34. Pell D, Penney T, Hammond D, Vanderlee L, White M, Adams J. Support for, and perceived effectiveness of, the UK soft drinks industry levy among UK adults: cross-sectional analysis of the International Food Policy Study. BMJ Open 2019;**9**:e026698.

35. Gillison F, Grey E, Griffin T. Parents' Perceptions and Responses to the UK Soft Drinks Industry Levy. J Nutr Educ Behav 2020;**52**:626-31.

36. HM Revenue & Customs. Soft Drinks Industry Levy 2016. https://www.gov.uk/government/publications/soft-drinks-industry-levy/soft-drinks-industry-levy. Accessed 5 December

37. Gauke D. Budget 2016. London: HM Treasury, 2016.

38. Scarborough P, Adhikari V, Harrington RA, et al. Impact of the announcement and implementation of the UK Soft Drinks Industry Levy on sugar content, price, product size and number of available soft drinks in the UK, 2015-19: A controlled interrupted time series analysis. PLoS Medicine 2020;**17**:e1003025.

39. Pell D, Mytton O, Penney T, et al. Changes in British household purchases of soft drinks associated with implementation of the Soft Drinks Industry Levy: a controlled interrupted time series analysis. BMJ 2021;**372**:doi:10.1136/bmj.n254.

40. The American Association for Public Opinion Research. Standard Definitions: Final Dispositions of Case Codes and

Outcome Rates for Surveys 2016. https://www.aapor.org/AAPOR_Main/media/publications/Standard-Definitions20169theditionfinal.pdf. Accessed 2 July 2021

41. HM Government. Childhood Obesity: A Plan for Action, 2016.

42. Litwin H, Sapir EV. Perceived Income Adequacy Among Older Adults in 12 Countries: Findings From the Survey of Health, Ageing, and Retirement in Europe. The Gerontologist 2009;**49**:397-406.

43. Vanderlee L, Reid JL, White CM, et al. Evaluation of the online Beverage Frequency Questionnaire (BFQ). Nutr J 2018;**17**:73.

For peer review only - http://bmjopen.bmj.com/site/about/guidelines.xhtml

44. Adams J, Bateman B, Becker F, et al. Effectiveness and acceptability of parental financial incentives and quasi-mandatory schemes for increasing uptake of vaccinations in preschool children: systematic review, qualitative study and discrete choice experiment. Health technology assessment (Winchester, England) 2015;**19**:1-176.

45. Vanderlee L, White CM, Kirkpatrick SI, et al. Non-alcoholic and alcoholic beverage intakes by adults across five upper-middle and high-income countries. Journal of Nutrition 2021;**151**:140-51.

46. Smith G. More than 300 businesses hit by sugar tax - but revenues will be half expected 2018. https://www.newfoodmagazine.com/news/72139/more-than-300-sugar-tax/. Accessed 10 Feb 2021

47. Buckton CH, Fergie G, Leifeld P, Hilton S. A discourse network analysis of UK newspaper coverage of the "sugar tax" debate before and after the announcement of the Soft Drinks Industry Levy. BMC Public Health 2019;**19**:490.

48. Buckton CH, Patterson C, Hyseni L, et al. The palatability of sugar-sweetened beverage taxation: A content analysis of newspaper coverage of the UK sugar debate. PLoS One 2018;**13**:e0207576.

49. Theis DRZ, White M. Is Obesity Policy in England Fit for Purpose? Analysis of Government Strategies and Policies, 1992-2020. The Milbank quarterly 2021;**99**:126-70.

For peer review only

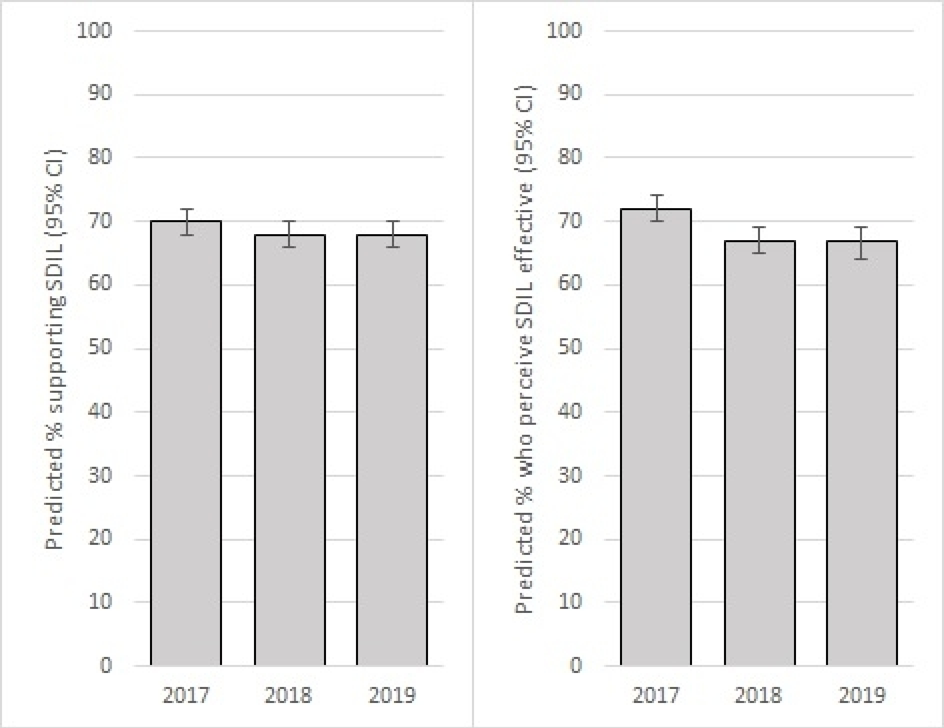

Predicted percentage (95% confidence intervals) of participants who supported (left) and perceived the Soft Drinks Industry Levy to be effective (right); International Food Policy Study, 2017-19

124x96mm (96 x 96 DPI)

STROBE Statement—Checklist of items that should be included in reports of ***cross-sectional studies***

	Item No	Recommendation	Page No
**Title and abstract**	1	(*a*) Indicate the study's design with a commonly used term in the title or the abstract	1
		(*b*) Provide in the abstract an informative and balanced summary of what was done and what was found	2
**Introduction**			
Background/rationale	2	Explain the scientific background and rationale for the investigation being reported	4-5
Objectives	3	State specific objectives, including any prespecified hypotheses	5
**Methods**			
Study design	4	Present key elements of study design early in the paper	6
Setting	5	Describe the setting, locations, and relevant dates, including periods of recruitment, exposure, follow-up, and data collection	6
Participants	6	(*a*) Give the eligibility criteria, and the sources and methods of selection of participants	6
Variables	7	Clearly define all outcomes, exposures, predictors, potential confounders, and effect modifiers. Give diagnostic criteria, if applicable	6-8
Data sources/ measurement	8*	For each variable of interest, give sources of data and details of methods of assessment (measurement). Describe comparability of assessment methods if there is more than one group	6-8
Bias	9	Describe any efforts to address potential sources of bias	6-9
Study size	10	Explain how the study size was arrived at	6
Quantitative variables	11	Explain how quantitative variables were handled in the analyses. If applicable, describe which groupings were chosen and why	6-9
Statistical methods	12	(*a*) Describe all statistical methods, including those used to control for confounding	9
		(*b*) Describe any methods used to examine subgroups and interactions	NA
		(*c*) Explain how missing data were addressed	7
		(*d*) If applicable, describe analytical methods taking account of sampling strategy	9
		(*e*) Describe any sensitivity analyses	NA
**Results**			
Participants	13*	(a) Report numbers of individuals at each stage of study—eg numbers potentially eligible, examined for eligibility, confirmed eligible, included in the study, completing follow-up, and analysed	9
		(b) Give reasons for non-participation at each stage	9
		(c) Consider use of a flow diagram	NA
Descriptive data	14*	(a) Give characteristics of study participants (eg demographic, clinical, social) and information on exposures and potential confounders	10
		(b) Indicate number of participants with missing data for each variable of interest	10
Outcome data	15*	Report numbers of outcome events or summary measures	10
Main results	16	(*a*) Give unadjusted estimates and, if applicable, confounder-adjusted estimates and their precision (eg, 95% confidence interval). Make clear which confounders were adjusted for and why they were included	12

For peer review only - http://bmjopen.bmj.com/site/about/guidelines.xhtml

		(*b*) Report category boundaries when continuous variables were categorized	7
		(*c*) If relevant, consider translating estimates of relative risk into absolute risk for a meaningful time period	NA
Other analyses	17	Report other analyses done—eg analyses of subgroups and interactions, and sensitivity analyses	NA
**Discussion**			
Key results	18	Summarise key results with reference to study objectives	13
Limitations	19	Discuss limitations of the study, taking into account sources of potential bias or imprecision. Discuss both direction and magnitude of any potential bias	13-14
Interpretation	20	Give a cautious overall interpretation of results considering objectives, limitations, multiplicity of analyses, results from similar studies, and other relevant evidence	14-16
Generalisability	21	Discuss the generalisability (external validity) of the study results	13
**Other information**			
Funding	22	Give the source of funding and the role of the funders for the present study and, if applicable, for the original study on which the present article is based	15

*Give information separately for exposed and unexposed groups.

Note: An Explanation and Elaboration article discusses each checklist item and gives methodological background and published examples of transparent reporting. The STROBE checklist is best used in conjunction with this article (freely available on the Web sites of PLoS Medicine at http://www.plosmedicine.org/, Annals of Internal Medicine at http://www.annals.org/, and Epidemiology at http://www.epidem.com/). Information on the STROBE Initiative is available at www.strobe-statement.org.

For peer review only
